# Viral cross-class transmission results in disease of a phytopathogenic fungus

**DOI:** 10.1038/s41396-022-01310-y

**Published:** 2022-08-31

**Authors:** Yue Deng, Kang Zhou, Mingde Wu, Jing Zhang, Long Yang, Weidong Chen, Guoqing Li

**Affiliations:** 1 State Key Laboratory of Agricultural Microbiology, Huazhong Agricultural University, Wuhan, 430070 China; 2 Hubei Key Laboratory of Plant Pathology, Huazhong Agricultural University, Wuhan, 430070 China; 3 U.S. Department of Agriculture, Agricultural Research Service, Washington State University, Pullman, WA 99164 USA

**Keywords:** Virology, Fungi

## Abstract

Interspecies transmission of viruses is a well-known phenomenon in animals and plants whether via contacts or vectors. In fungi, interspecies transmission between distantly related fungi is often suspected but rarely experimentally documented and may have practical implications. A newly described double-strand RNA (dsRNA) virus found asymptomatic in the phytopathogenic fungus *Leptosphaeria biglobosa* of cruciferous crops was successfully transmitted to an evolutionarily distant, broad-host range pathogen *Botrytis cinerea*. Leptosphaeria biglobosa botybirnavirus 1 (LbBV1) was characterized in *L. biglobosa* strain GZJS-19. Its infection in *L. biglobosa* was asymptomatic, as no significant differences in radial mycelial growth and pathogenicity were observed between LbBV1-infected and LbBV1-free strains. However, cross-species transmission of LbBV1 from *L. biglobosa* to infection in *B. cinerea* resulted in the hypovirulence of the recipient *B*. *cinerea* strain t-459-V. The cross-species transmission was succeeded only by inoculation of mixed spores of *L*. *biglobosa* and *B*. *cinerea* on PDA or on stems of oilseed rape with the efficiency of 4.6% and 18.8%, respectively. To investigate viral cross-species transmission between *L*. *biglobosa* and *B*. *cinerea* in nature, RNA sequencing was carried out on *L*. *biglobosa* and *B*. *cinerea* isolates obtained from *Brassica* samples co-infected by these two pathogens and showed that at least two mycoviruses were detected in both fungal groups. These results indicate that cross-species transmission of mycoviruses may occur frequently in nature and result in the phenotypical changes of newly invaded phytopathogenic fungi. This study also provides new insights for using asymptomatic mycoviruses as biocontrol agent.

## Introduction

Virulence is a key trait of pathogens, but some scientists believe that virulence brings very few benefits for pathogens, unless it is necessary for their transmission [1]. According to “conventional wisdom”, parasites should evolve to be increasingly benign or less virulent, as diseases can decrease the fitness of hosts and may lead to the extinction of possibly both the hosts and the microbes [2–4]. However, the evolution of virulence is also influenced by many factors, such as host condition [5, 6], host density [7, 8], or interactions with other species [9]. In some cases, increased virulence may be accompanied with host shift [10], changes in host population [11], or the competition with other parasites [12]. For instance, the dense and uniform host populations in an agro-ecosystem favor the emergence of highly virulent, host-specialized plant pathogens, and this is an important factor driving the origins of plant pathogens in agro-ecosystems [13]. In contrast, long-term co-evolution between hosts and pathogens may favor decreased virulence [14, 15], in this case, mycoviruses are considered to be a good example [16, 17].

Mycoviruses are viruses infecting fungi, yeasts, and oomycetes, which are ubiquitous in all major fungal groups. They were first reported in 1962 causing a disease of the cultivated button mushroom *Agaricus bisporus* [18]. Since then, a large number of mycoviruses have been discovered, and most of them have double-strand (ds) or single-strand (ss) RNA genomes, while a few possess genomes of DNA. In some cases, mycoviruses can significantly alter the biology of their hosts, such as reduction of mycelial growth rate and attenuation of virulence (e.g., hypovirulence). In contrast, some others are able to enhance the virulence of their fungal hosts [19–23]. Furthermore, mycoviruses can participate in some complex mutualistic symbiosis, enhancing the survival of host fungi and plants under extreme conditions [24]. In addition, mycoviruses may also be involved in the interaction between biocontrol agents and target phytopathogenic fungi [25]. Although mycoviruses having biological effects are frequently observed and widely investigated, most mycoviral infections are asymptomatic or cryptic, and this may be related to their lifestyle. Different from animal or plant viruses, mycoviruses are deficient of extracellular stage in their lifecycles [26], with a few exceptions [27]. Most of them are still believed to be mainly transmitted vertically through spores and horizontally via hyphal anastomosis between vegetatively compatible individuals [28]. As a consequence, their lack of virulence may be due to their limited transmission among different individuals, therefore, in the long run, mycoviral infection is likely to be relatively benign, or possibly even beneficial [3, 4, 16, 29–32].

Although most mycoviral infections are asymptomatic, hypovirulence-associated mycoviruses are widely reported in many phytopathogenic fungi, and they are also the most investigated viral group [33, 34], as some of them could be used or have the potential for the control of plant fungal diseases [35]. This raises the question that why this viral group is widely detected in different fungal species, as this is contrary to the above-mentioned arguments. Moreover, many hypovirulent fungal strains carrying mycoviruses can be initially isolated from diseased plants with typical symptoms. These strains were less virulent or even avirulent on plants when being re-inoculated on the same host plants, and some biological characteristics may change as well, such as sporulation and sclerotial formation [36, 37]. If these strains were hypovirulent or avirulent at the beginning, how could these strains cause severe damage on plants? To answer this question, one explanation is that these fungal strains may have acquired the mycoviruses recently between species [38]. Similar cases have been widely investigated in animal viruses [39].

Compared with plant and fungal viruses, animal viruses are the most intensively studied viral group, as some of them can cause infectious diseases that threaten humans and animals [40–42]. Although some animal viruses have a wide host range, they do not cause significant symptoms in all hosts [43, 44], and one typical example is bat viruses. Due to recent studies of virus tracing, bats are considered as reservoirs for many emerging zoonotic viruses [45], including rabies virus [46], Nipah virus [47], Hendra virus [48], Marburg virus [49], Zaire Ebolavirus [50], and coronaviruses such as severe acute respiratory syndrome coronavirus (SARS-CoV) [40, 51], Middle East respiratory coronavirus [52], and recently discovered SARS-CoV-2 which causes the current pandemic of COVID-19 [53]. Although bats harbor so many zoonotic viruses, most of them do not seem to show ostensible symptoms [54].

**Fig. 1:**
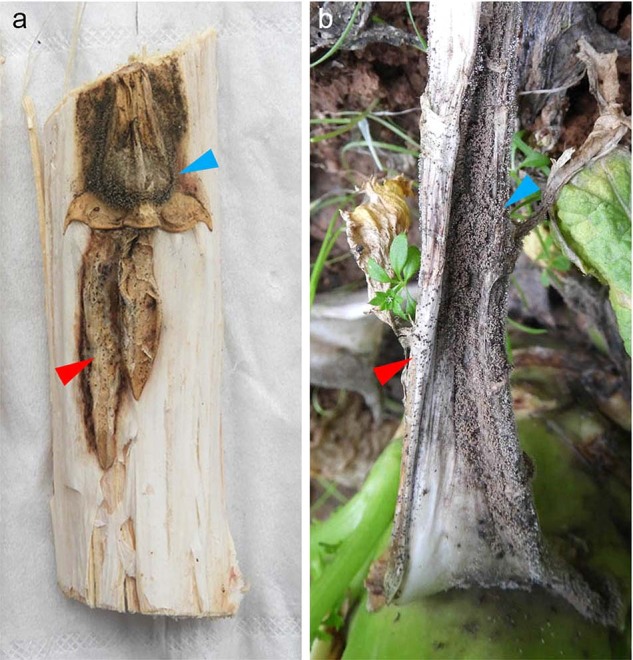
Field symptoms of *Brassica* crops co-infected by *Botrytis cinerea* and *Leptosphaeria biglobosa*. **a** Co-infection of two pathogens on stem of *Br. napus* and **b** co-infection of two pathogens on petiole of *Br. juncea* var. *tumida*. Red and blue arrowheads indicate the presence of conidia of *B*. *cinerea* and pycnidia of *L*. *biglobosa* on plant tissues, respectively.

To test whether similar situation occurs in fungi, we investigated the possibility of viral transmission between two fungal species, *Leptosphaeria biglobosa* and *Botrytis cinerea*, which have similar ecological niches on oilseed rape (*Brassica napus* L.). *L. biglobosa*, together with its sister species *L. maculans*, is the causative agent of black leg disease in *Br. napus* and many other *Brassica* species worldwide [55]. Both two fungal pathogens could cause lesions on leaves or cankers on stems, of which *L. maculans* is more aggressive than *L. biglobosa*. These two related species co-exist in most *Br. napus* growing regions of the world except in China where only *L. biglobosa* has been reported. *B*. *cinerea* has a wide host range including more 1400 plant species, and causes great losses on many economically important crops [56]. Similarly, oilseed rape and other *Brassica* vegetables are also ideal hosts for *B*. *cinerea*. Both fungal pathogens infect similar tissues of *Brassica* crops, including leaves and stems. Sometimes, co-infection of two fungi on the same plant tissue could be observed (Fig. 1). Therefore, we speculate that cross-species transmission of mycoviruses may occur between the two species.

In the present study, a mycovirus namely Leptosphaeria biglobosa botybirnavirus 1 (LbBV1) was used for testing viral cross-species transmission between *L*. *biglobosa* and *B*. *cinerea*. We first characterized LbBV1, and investigated the effects of LbBV1 on its native host *L. biglobosa*. Then, the transmission of LbBV1 from *L*. *biglobosa* to *B*. *cinerea* was also tested, as well as the biological changes mediated by LbBV1 on *B*. *cinerea*. Moreover, viral cross-species transmission between *B*. *cinerea* and *L*. *biglobosa* under field conditions was investigated by using high-throughput sequencing. Our results indicate that viral cross-species transmission may occur frequently in nature if two fungal species share the same ecological niche, and this may lead to hypovirulence of newly invaded phytopathogenic fungi.

## Materials and methods

### Fungal strains and biological characterization

*L. biglobosa* strain GZJS19 was originally isolated from diseased oilseed rape stem with “blackleg” symptom collected in Jinsha County, Guizhou Province, China following the method previously described [57]. All fungal strains (Table S1) were cultured on potato dextrose agar (PDA) at 20 °C and stored at 4 °C, or fungal mycelial plugs were stored in 20% sterilized glycerol at −80 °C. To remove LbBV1 from strain GZJS19, the hyphal tips of strain GZJS19 were picked out and cultured on PDA for ten generations, and a LbBV1-free strain GZJS19-VF was obtained.

*B. cinerea* and *L. biglobosa* strains on PDA were incubated at 20 °C (12 h light/12 h dark) for determination of mycelial growth rate and for observation of colony morphology. Virulence assay of *L. biglobosa* on *Br. napus* (20 °C, 7 day) and *B. cinerea* on *Nicotiana benthamiana* (20 °C, 72 h) were performed according to established protocols [58, 59]. The radial mycelial growth rate of two fungal species on PDA was determined using the procedures described in our previous studies [59].

### DNA Extraction and fungal strain identification

Total DNA of fungal mycelia grown on PDA was extracted by using the cetyltrimethylammonium bromide method. The simple sequence repeat (SSR) analysis was used to distinguish different *L. biglobosa* strains with the primer pair SSR17-F/SSR17-R (Table S2). The identities of *L. biglobosa* and *B. cinerea* strains were confirm by using PCR with species-specific primer pairs LbigF/LmacR and Bc-F/Bc-R (Table S2), respectively.

### RNA extraction, viral genome sequencing, and RT-PCR detection

Extraction and purification of dsRNA and total RNA from mycelia of *B*. *cinerea* and *L. biglobosa* were performed as described previously [60]. The dsRNA was further confirmed based on the resistance to DNase Ι and S1 nuclease (Promega, Madison, WI, USA). All RNA samples were stored at −80 °C, and was fractionated by agarose gel (1%, *ѡ*/*ν*) electrophoresis and visualized by staining with ethidium bromide (1.5 μg/L) and viewing on a UV trans-illuminator.

To determine the genome of LbBV1, the purified dsRNAs were excised from the gel and purified using AxyPrepTM DNA Gel Extraction Kit (Axygen Scientific, Inc.; Union City, CA, USA). The cDNA library of two dsRNA bands was produced using a random primer-mediated PCR amplification protocol [61] and sequenced as previously described [62]. The terminal sequences of each dsRNA were cloned through ligating the 3’-terminus for each strand of each dsRNA with the 5′-terminus of the 110A adaptor (Table S3) using T4 RNA ligase (Promega Corporation, Madison, WI, USA) at 16 °C for 18 h, and then reverse transcribed using primer RC110A (Table S3). All these amplicons were detected by agarose gel electrophoresis, gel-purified, and cloned into *E*. *coli* DH5α, sequenced, and assembled as previously described [61] to obtain the full-length cDNA sequences of LbBV1.

For reverse transcription-PCR (RT-PCR) detection of LbBV1, cDNA was synthesized using Reverse Transcriptase M-MLV (RNAse H-) and two pairs of specific primers (RT-1-F/T-1-R, RT-2-F/RT-2-R, Table S3), which were designed based on the sequences of dsRNA-1 and dsRNA-2, generating amplicons of 871 bp and 870 bp in size, respectively. PCR amplification was carried out by using 2× PCR Mix (Tsingke Biotechnology Co., Ltd.) with the above specific primer pairs.

### Sequence analysis of LbBV1

The full-length cDNA sequences of two dsRNAs were used as queries to BLAST search the public database at National Center for Biotechnology Information (NCBI, https://www.ncbi.nlm.nih.gov). ORFs were deduced using the ORF Finder program in NCBI (http://www.ncbi.nlm.nih.gov/gorf/gorf.html). Conserved domains of each dsRNA were deduced using CDD database (http://www.ncbi.nlm.nih.gov/ Structure/cdd/wrpsb.cgi). Multiple alignments of the sequences of RNA-dependent RNA polymerase (RdRp) were accomplished using the DNAMAN program and phylogenetic trees based on the sequences of RdRp domain were constructed using the neighbor-joining (NJ) method and tested with a bootstrap of 1000 replicates to determine the reliability of a given branch pattern in MEGA 7.0.

### Purification of viral particles and TEM

Viral particles were purified as described previously [62] with some modifications. Mycelial plugs of strain GZJS19 were inoculated in potato dextrose broth in shake culture 150 rpm/min at 20 °C for 7 days. Mycelia were harvested by filtration with three layers of lens tissue. About 30 g of mycelia were ground into fine powder in liquid nitrogen, and then transferred to a 1000 ml-blender containing 200 ml extraction solution of 0.1 M sodium phosphate with 3% (wt/vol) Triton X-100 (pH 7.0). The mixture was blended twice for 2 min each. Then, the suspension was transferred to 50 ml plastic tubes, followed by centrifugation at 10,000 × *g* for 20 min to remove the hyphal cell debris. The supernatant was then carefully pipetted out and ultracentrifuged at 119,000 × *g* under 4 °C for 2 h. The pellet was re-suspended in 0.5 ml of sodium phosphate buffer (0.05 M), and the re-suspended solution was then overlaid on a centrifuge tube containing sucrose solutions with a concentration gradient ranging from 10 to 50% (wt/vol), and centrifuged at 70,000 × *g* under 4 °C for 2 h.

Each fraction was carefully collected and suspended in 150 μl of 0.05 M sodium phosphate buffer (pH 7.0) and was individually tested for the presence of dsRNA by agarose gel electrophoresis. The purified virus particles were stained with phosphotungstic acid solution (20 g/l [wt/vol], pH 7.4) and observed under a transmission electron microscopy (TEM, Hitachi HT7800/HT7700). The structure proteins from viral particles were detected by electrophoresis on a 12% (wt/vol) polyacrylamide gel (PAGE) amended with 1% (wt/vol) sodium dodecyl sulfate (SDS).

### Northern hybridization

Northern hybridization was performed to confirm the authenticity of the cDNA sequences generated from dsRNA-1 (6.2 kb) and dsRNA-2 (5.9 kb) in strains GZJS19 of *L. biglobosa* and t-459-V of *B. cinerea*. Two DNA probes, nt positions 1228–1697 for Probe 1 and nt positions 2318–2957 for Probe 2 (Table S3), were designed based on full-length cDNA sequences of dsRNA-1 and dsRNA-2, respectively. The purified dsRNA-1 and dsRNA-2 were separated in 1% (w/v) agarose gel and transferred to Immobilon-Ny^+^ membranes (Millipore, Bedford, MA, USA) by the capillary transfer method using 20 × SSC as transfer buffer [62]. Probe 1 and Probe 2 were pre-labeled as described by the manufacturers (GE Healthcare, Little Chalfont, United Kingdom) for hybridization with the denatured dsRNAs blotted on two membranes, respectively. The chemiluminescent signals of the probe-RNA hybrids were detected by using a CDP-Star kit (GE Healthcare Life Sciences, China).

### Vertical transmission of LbBV1 in *L*. *biglobosa*

Vertical transmission refers to transmission of hypovirulence-associated dsRNAs from mycelia to the next generation via asexual or sexual spores [33]. Here, vertical transmission of LbBV1 refers to viral transmission through the asexual spore—conidia of *L*. *biglobosa*. Conidial suspension (1 × 10^2^ conidia/ml) was evenly spread on PDA plates (150 μl/plate) for the generation single conidial colonies. All single conidial (SC) strains were subjected to LbBV1 detection with RT-PCR. Six SC strains (VT-1, VT-2, VT-3, VT-4, VT-5, and VT-6) were selected for investigation of biological property.

### Intra- and interspecies transmission of LbBV1

For intraspecies transmission, the pairing culture technique as previously described [63] was used to test the viral transmission from *L. biglobosa* strain GZJS19 to strains Lb731 and W10, and from LbBV1 infected derivative strain HT-2 to W10. Strain W10 was resistant to hygromycin, which could be used to rule out the contamination by strain GZJS19 as both strains shared the same SSR marker. The derivative strains of W10 were cultured on PDA containing hygromycin B (50 μg/ml). For interspecies transmission, three methods were explored to test the viral transmission from *L. biglobosa* strain GZJS19 to *B*. *cinerea* strain t-459. Besides pairing culture technique, conidia of strain GZJS19 and t-459 were mixed and co-cultured on PDA to evaluate the efficiency of interspecies transmission of LbBV1. Conidial suspension of *B*. *cinerea* strain t-459 (200 μl, 1 × 10^5^ conidia/ml) was mixed with that of *L. biglobosa* strain GZJS19 (800 μl, 1 × 10^5^ conidia/ml), and 100 μl of the mixed conidial suspension were evenly spread on a PDA plate with ten repeats and inoculated at 20 °C for 24 h (Fig. S1). Moreover, mixed conidial suspension of the same composition was also inoculated on sterilized healthy oilseed rape stems (8 repeats) in a sealed flask at 20 °C for 2 weeks (Fig. S2). The presence of LbBV1 in all derivative strains and biological properties of partial derivative strains were determined as described above.

### Protoplast transfection

Protoplast transfection was used to introduce viral particle of LbBV1 into protoplast of *B*. *cinerea* strain t-459 in the presence of polyethylene glycol (PEG) 4000 [63]. LbBV1 particles were mixed gently with 100 μl t-459 protoplast suspension and inoculated in ice bath for 30 min, then 200 μl, 200 μl, and 600 μl PEG solutions were gradually added to the above-mixed suspension in three steps and inoculated at 20 °C for 20 min. The mixed suspension of 200 μl was evenly mixed with 20 ml regeneration medium and then poured in six Petri plates and inoculated at 20 °C for 2 days. Single regenerated colonies were picked up from the surface of regeneration medium and subsequently cultured before detection of LbBV1.

### High through-put sequencing and sequence analysis

To test whether viral cross-species transmission occurs between *B. cinerea* and *L. biglobosa* under natural conditions, two fungal species from same infected tissues of *Brassica* crops were isolated and used for RNA-seq. Sixteen *B. cinerea* strains and 15 *L. biglobosa* strains (Table S4) were finally obtained. Total RNA samples were extracted as described above. One microgram of RNA was taken from each strain, and RNA from two fungal species were then mixed respectively into two samples (*B. cinerea* and *L. biglobosa*) and sent to GENEWIZ (Suzhou, China) for RNA sequencing. Ribosomal RNA depletion (Ribo-Zero rRNA Removal Kit, Illumina, Inc.), library preparations (~1 μg RNA, TruSeq RNA Sample Preparation Kit, Illumina, Inc.), and high-throughput sequencing in a HiSeq X ten system (Illumina, Inc.) were accomplished by GENEWIZ. The unqualified reads were filtered out, then clean reads were spliced from scratch using software Trinity (version: 2.3.3), and the resulting sequences were then deduplicated with cd-hit (version: 4.6). Finally, the software Diamond (version: 0.8.22) and the non-redundant protein database in the National Center for Biotechnology Information (NCBI) (https://www.ncbi.nlm.nih.gov/) were used for BLASTx annotation, and the viral sequences were selected. The presence of each virus was also confirmed by using RT-PCR with specific primers designed according to the viral sequences.

### Statistical analysis

Fungal mycelial growth rate and lesion diameter of strains of *L. biglobosa* and *B. cinerea* were analyzed by using analysis of variance in SAS V8.0 (SAS Institute, Cary, NC, USA, 1999). Treatment means on each of these two parameters for the tested strains or isolates were compared using the least-significant-difference test at *α* = 0.05.

## Results

### Viral genome and particle of LbBV1

**Fig. 2:**
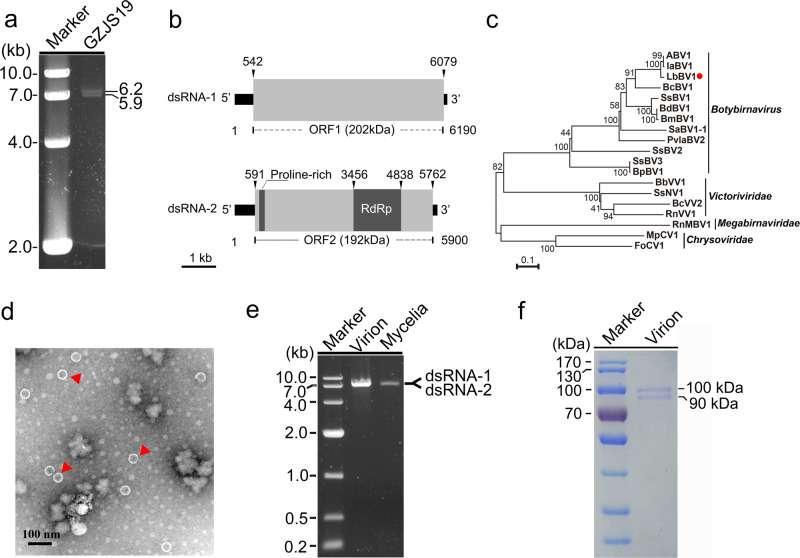
Genomic organization, virus particles, viral dsRNAs, and viral structure proteins of Leptosphaeria biglobosa botybirnavirus 1 (LbBV1). **a** Agarose gel electrophoresis of dsRNAs extracted from the mycelium of *Leptosphaeria biglobosa* strain GZJS19. Marker, DNA marker D10000 (TaKaRa). **b** Schematic diagram of the genetic organization of LbBV1. The coding strand of dsRNA-1 is 6190 bp long and comprises one large ORF, designated ORF 1, which encodes a polyprotein with a calculated molecular mass of 202 kDa. The coding strand of dsRNA-2 is 5900 bp long and also comprises one large ORF, designated ORF 2, which encodes a polyprotein with a calculated molecular mass of 192 kDa. **c** Phylogenetic analysis of the RNA-dependent RNA polymerase (RdRp) region of LbBV1 and selected dsRNA viruses of *Chrysoviridae*, *Megabirnaviridae*, “*Botybirnavirus*” and *Victoriviridae*. The red dot denotes the position of LbBV1. Numbers at the nodes indicate the bootstrap values out of 1000 replicates. **d** Transmission electron microscopy (TEM) images of the virus particles (~37 nm in diameter) purified from *L. biglobosa* strain GZJS19. Arrowheads indicate the virus particles. **e** Agarose gel electrophoresis analysis of the dsRNAs extracted from purified virus particles of LbBV1 and the mycelia of *L. biglobosa* strain GZJS19. Note that the two dsRNAs of LbBV1 were not separated. Marker, DNA marker D10000 (TaKaRa). **f** SDS-PAGE analysis of structural proteins extracted from purified virus particles of LbBV1.

LbBV1 has two dsRNA segments, namely dsRNA-1 and dsRNA-2, with the sizes of 6190 and 5900 bp, respectively (Fig. 2a). Moreover, the nucleotide sequences of dsRNA-1 and dsRNA-2 at the 3′-termini (80 bp) and the 5′-termini (500 bp) shared sequence identities of 57% and 84%, respectively (Fig. S3). Each dsRNA segment contained one large open reading frames (ORF), namely ORF1 and ORF2, putatively encoding two polypeptides of 202 and 192 kDa for dsRNA 1 and dsRNA 2, respectively (Fig. 2b). The ORF2-encoded polypeptide contained a proline-rich region and an RdRp domain (Fig. 2b). The cDNA sequences of LbBV1 were most related to those of Alternaria botybirnavirus 1 (ABV1) with the identity of 78% and 81% to dsRNA-1 and dsRNA-2, respectively. Moreover, they also showed relative high sequence similarity to the dsRNA-2 and dsRNA-1 of Botrytis cinerea botybirnavirus 1 (BcBV1) with the nucleotide identity of 56% and 48%, respectively. The eight conserved motifs (I–VIII) botybirnaviral RdRp sequences were also detected (Fig. S4). Therefore, this virus was named as Leptosphaeria biglobosa botybirnavirus 1 (LbBV1). Phylogenetic analysis showed that LbBV1 and ABV1 formed a clade with the bootstrap of 100%, then clustered with BcBV1, and finally formed an independent clade together with other botybirnaviruses with the bootstrap of 100% (Fig. 2c). Viral particles purified from strain GZJS19 of *L. biglobosa* were isometric and ~37 nm in diameter (Fig. 2d), accommodating two dsRNA segments with the sizes similar to those isolated from the mycelia of strain GZJS19 (Fig. 2e). SDS-PAGE analysis of the purified particles revealed the presence of two major structure proteins with estimated molecular weight of about 100 and 90 kDa, respectively (Fig. 2f).

### Biological properties of *L*. *biglobosa* strain GZJS19

**Fig. 3:**
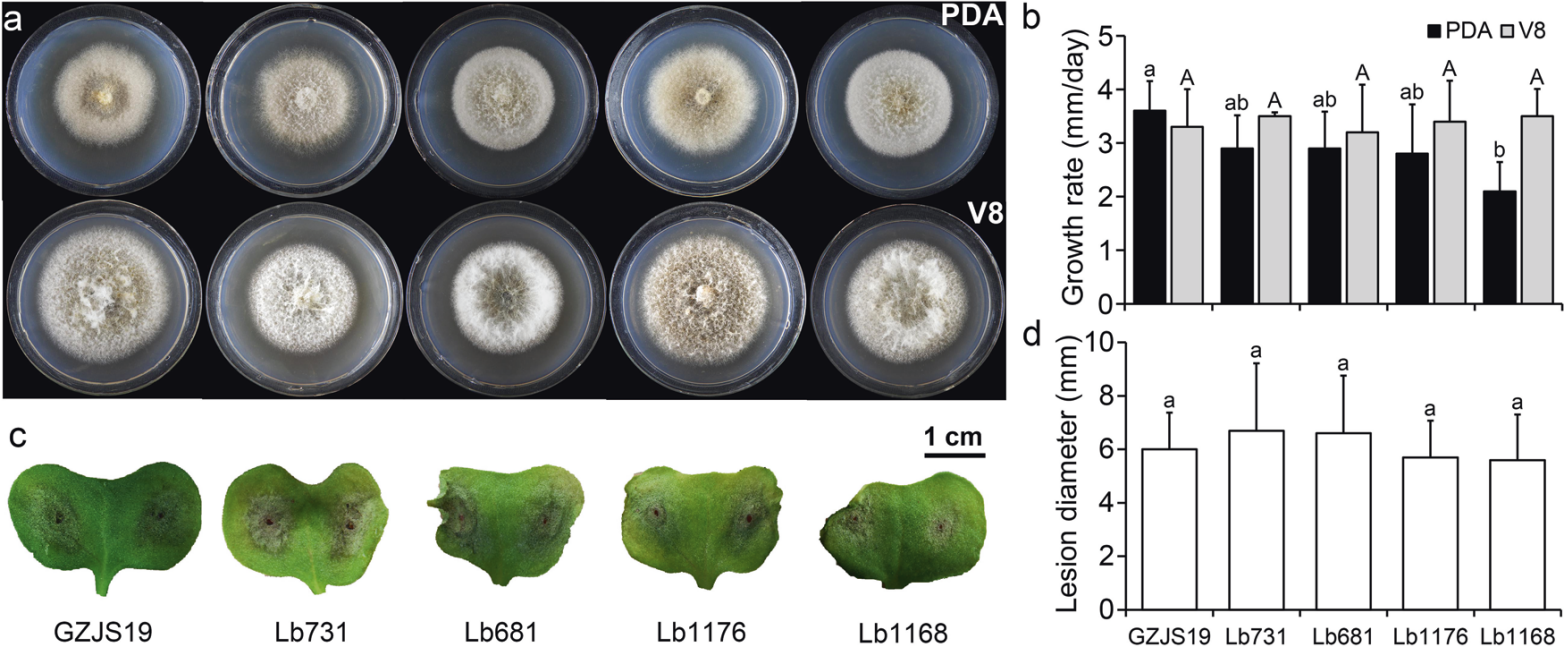
Biological properties of different *Leptosphaeria biglobosa* strains isolated from the fields. **a** Colony morphology (23 °C, 8 days) of strains GZJS19, Lb731, Lb681, Lb1176, and Lb1168 on potato dextrose agar (PDA) and V8 juice agar (V8). **b** Radial mycelial growth rate (23 °C) on PDA and V8. **c** Pathogenicity assay (23 °C,7 days) of strains GZJS19, Lb731, Lb681, Lb1176, and Lb1168 on cotyledon of oilseed rape. **d** Lesion diameter (23 °C, 7 days) on cotyledon of rapeseed of strains GZJS19, Lb731, Lb681, Lb1176, and Lb1168. The results are expressed as arithmetic means the standard errors of the means. In each histogram, bars labeled with the same letters are not significantly different (*p* > 0.05) according to the least-significant-difference test.

Four field strains of *L. biglobosa* free of LbBV1 isolated from diseased *Br*. *napus* were used for biological property analysis together with strain GZJS19 to investigate the biological effect of LbBV1. The results showed that strain GZJS19 produced fluffy white aerial mycelia and lots of pycnidia on both PDA and V8 plates (20 °C, 7 d), which is similar to the other four LbBV1-free strains (Fig. 3a). Moreover, the radial mycelial growth of strain GZJS19 on PDA and V8 was also comparable to the other four LbBV1-free strains (Fig. 3b). The pathogenicity assays showed that all strains of *L*. *biglobosa* could cause necrotic lesions on cotyledons of *Br*. *napus*, and the average lesion size caused by strain GZJS19 was not significantly different from those of the other four LbBV1-free strains (Fig. 3c, d). Furthermore, the LbBV1-free strain GZJS19-VF also showed similar radial mycelial growth and virulence to those of strain GZJS19, although the fungal colony of strain GZJS19-VF was slightly darker than that of strain GZJS19 (Fig. S5). Overall, LbBV1 did not cause any significant phenotypical changes, especially on mycelial growth and virulence, in *L*. *biglobosa*, compared to the LbBV1-free strains.

### Vertical and horizontal transmission of LbBV1

All 42 single conidium strains from the strain *L. biglobosa* GZJS19 carried LbBV1 by using RT-PCR detection (Fig. S6) and the 6 arbitrarily selected strains (VT-1, VT-2, VT-3, VT-4, VT-5, and VT-6) showed similar biological properties to those of strain GZJS19 (Fig. S7). These results suggested that LbBV1 could be efficiently transmitted vertically through conidia of *L*. *biglobosa* without causing any visible changes.

**Fig. 4:**
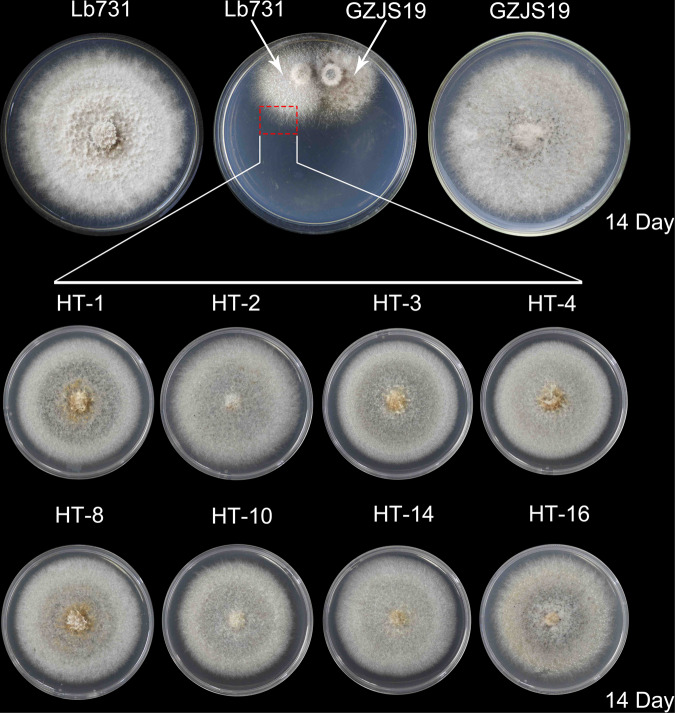
Horizontal transmission of LbBV1 from *Leptosphaeria biglobosa* strain GZJS19 to strain Lb731 by using pairing culture technique. The mycelium in the area of the dashed box were picked out to establish the derivative strains. The colony morphology (23 °C, 14 days) on potato dextrose agar of eight derivative strains used for biological property assays.

**Fig. 5:**
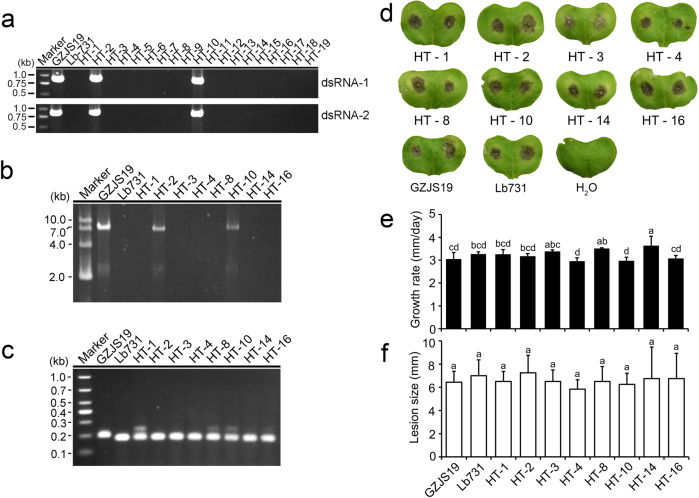
Biological properties of derivative *Leptosphaeria biglobosa* strains obtained from the colony of strain Lb731 after pair-culturing with strain GZJS19. **a** RT-PCR detection of dsRNA-1/dsRNA-2 presence in all derivative strains by using specific primers. **b** Dectection of dsRNAs extracted from the mycelia all derivative strains. Marker, DNA marker D10000 (TaKaRa). **c** Simple sequence repeats (SSR) analysis of strains GZJS19, Lb731, and all derivative strains by using agarose gel electrophoresis. **d** Pathogenicity assay (23 °C, 7 days) of strains GZJS19, Lb731, and derivative strains (HT-1, HT-2, HT-3, HT-4, HT-8, HT-10, HT-14, and HT-16) on cotyledons of oilseed rape. **e** Radial mycelial growth rate (23 °C) of strains GZJS19, Lb731, and derivative strains on PDA. **f** Lesion diameter (23 °C, 7 days) of strains GZJS19, Lb731, and derivative strains on cotyledons of oilseed rape. The results are expressed as arithmetic means the standard errors of the means. In each histogram, bars labeled with the same letters are not significantly different (*p* > 0.05) according to the least-significant-difference test.

Nineteen derivative strains were obtained from the colonies of strain Lb731 of *L. biglobosa* after paring culture with strain GZJS19 for 14 days (Fig. 4). Two of the 19 derivative strains (HT-2 and HT-10) were infected by LbBV1 based on dsRNA detection and RT-PCR detection (Fig. 5a, b). The analysis of simple sequence repeat (SSR) confirmed that the genetic backgrounds of 8 derivative strains were consistent with that of strain Lb731, not of strain GZJS19 (Fig. 5c). Biological property tests showed that no significant difference in colony morphology (20 °C, 7 d) was observed between virus-infected and virus-free strains. Similarly, virus-infected and virus-free strains also showed similar radial mycelial growth and virulence (Fig. 5d, e, f). In addtion, a LbBV1-free *L*. *biglobosa* strain W10 was used to compare the efficiency of LbBV1 horizontal transmission between *L*. *biglobosa* strains GZJS19 and HT-2 with different genetic background. Twenty derivative strains were obtained from the colonies of strain W10 after paring culture with strain GZJS19 and HT-2 for 14 days, respectively (Fig. S8a). Five of 20 and two of 20 derivative strains were infected by LbBV1 based on RT-PCR detection, respectively (Fig. S8b). These results indicated that LbBV1 could be horizontally transmitted to different *L*. *biglobosa* strains and LbBV1 are asymptomatic in *L*. *biglobosa* strains with different genetic backgrounds.

### Infection of LbBV1 conferred hypovirulence in *B*. *cinerea*

**Fig. 6:**
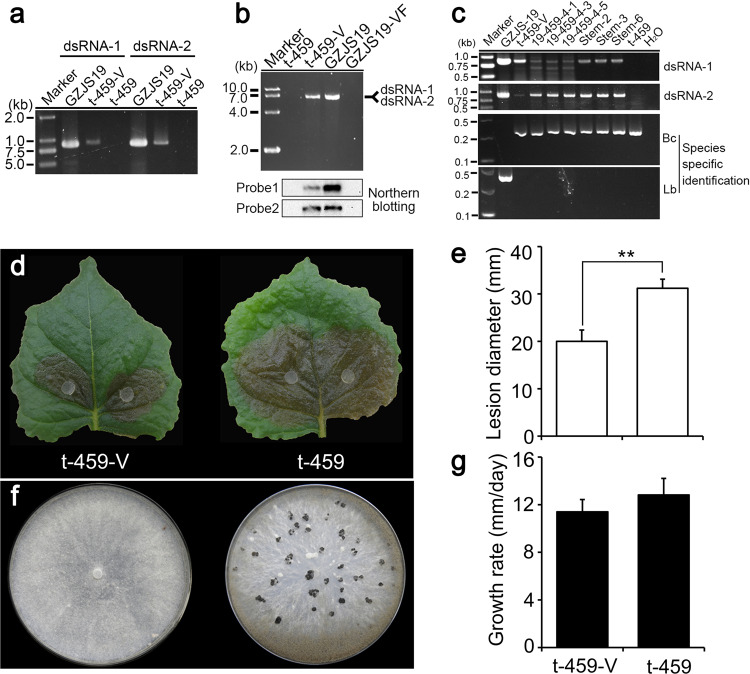
Biological effects of Leptosphaeria biglobosa botybirnavirus 1 (LbBV1) on Botrytis cinerea strain t-459. **a** RT-PCR detection of dsRNA-1/dsRNA-2 presence in t-459-V with specific primers. **b** Agarose gel electrophoresis and northern blotting detection of dsRNAs extracted from the mycelia of *L. biglobosa* strains (GZJS19 and GZJS19-VF) and *B. cinerea* strains (t-459-V and t-459). **c** RT-PCR detection of the presence of dsRNA-1/dsRNA-2 in *L*. *biglobosa* and *B*. *cinerea* strains obtained from the co-culture and co-inoculation tests of *L*. *biglobosa* strain GZJS19 and *B*. *cinerea* strain t-459. The identities of these strains were confirmed by using PCR with species-specific primers, Bc = *B*. *cinerea*, Lb = *L*. *biglobosa*. Marker, DNA marker D10000 (TaKaRa). **d** Pathogenicity assay (20 °C, 3 days) of strains t-459 and t-459-V on detached *Nicotiana benthamiana* leaves. **e** Lesion diameter (20 °C, 3 days, lower) on detached *N*. *benthamiana* leaves of strains t-459 and t-459-V. “**” indicates a significant difference (*p* < 0.01) between strains t-459 and t-459-V in pathogenicity. **f** Colony morphology (20 °C, 20 days) of strains t-459 and t-459-V on potato dextrose agar (PDA). **g** Radial mycelial growth rate (20 °C, upper) of strains t-459 and t-459-V on PDA.

Virus particles of LbBV1 were successfully introduced into protoplasts of *B. cinerea* strain t-459, and one LbBV1-infected strain t-459-V was obtained. Three lines of evidence suggested that LbBV1 was successfully introduced to *B. cinerea* strain t-459. First, two specific PCR products corresponding to dsRNA-1 and dsRNA-2 of LbBV1 were detected in *B*. *cinerea* strain t-459-V as well as in *L*. *biglobosa* strain GZJS19, but not in *B*. *cinerea* strain t-459 (Fig. 6a). Second, two LbBV1 dsRNA segments with the same sizes were detected in both strains t-459-V and GZJS19 (Fig. 6b). Finally, northern blotting confirmed the authenticity of LbBV1 presence in strains t-459-V as well as in strain GZJS19 (Fig. 6b). In addition, the species specific PCR products of *L. biglobosa* (441 bp) and *B. cinerea* (327 bp) confirmed that all cultures were pure (Fig. 6c). The accumulation of LbBV1 appeared to be less in *B*. *cinerea* than in *L*. *biglobosa* (Fig. 6a, b). Furthermore, we found the ratios of dsRNA-1 and dsRNA-2 in *B*. *cinerea* and *L*. *biglobosa* were different, of which dsRNA-1 accumulated less in *B*. *cinerea* than in *L*. *biglobosa* (Fig. 6b). Generally, LbBV1 could replicate stably in *B*. *cinerea* strain t-459-V, as it could be stably detected after subculture six times continuously. Different from the asymptomatic nature of LbBV1 infection in *L*. *biglobosa*, LbBV1 infection in *B*. *cinerea* resulted in some phenotypical changes. Pathogenicity assay on detached *N*. *benthamiana* leaves showed that the average lesion diameter of strain t-459-V (20 mm) was significant smaller than that of strain t-459 (30 mm; Fig. 6d, e). Moreover, strain t-459-V also lost the ability to produce sclerotia on PDA (20 °C, 20 d) in comparison with strain t-459 (Fig. 6f). Although not statistically different, the average radial mycelial growth rate of strain t-459-V (11 mm/d) was slightly slower than that of strain t-459 (12 mm/d) (Fig. 6g).

### Cross-species transmission of LbBV1

In the co-culture of conidia of both *L. biglobosa* strain GZJS19 and *B. cinerea* strain t-459 on PDA (Fig. S1), 65 derivative strains of *B*. *cinerea* were obtained and then subcultured on PDA for three generations. All these strains were detected for the presence of LbBV1 by RT-PCR using a specific primer pair RT-1-F/RT-1-R, and only 3 of 65 derivative strains (19-459-4-1, 19-459-4-3, and 19-459-4-5) showed the presence of LbBV1 (Fig. S1). Similarly, 16 derivative strains of *B. cinerea* were obtained from the parts of plant tissues showing the presence of conidiophores of *B*. *cinerea* after co-inoculation with conidia of both strains GZJS19 and t-459 (Fig. S2). In these derivative strains, three strains (Stem-2, Stem-3, and Stem-6) showed the presence of LbBV1 (Fig. S2). In addition, the identities of all derivative strains were also confirmed by using PCR with species-specific primers of *L. biglobosa* and *B. cinerea*, respectively (Fig. 6g). The stability of LbBV1 in these strains was also determined by using RT-PCR after sub-culturing on PDA for five generations, and LbBV1 could be detected in all derivative strains (Fig. 6g). Moreover, all these strains showed similar culture morphology to strain t-459-V, like the deficiency of sclerotium formation. However, the transmission of LbBV1 was not successful from *L*. *biglobosa* strain GZJS19 to *B*. *cinerea* strain t-459 by using paring culture technique, as no LbBV1 was detected in all 50 derivative strains.

### Possible mycoviral cross-species transmission in nature

**Fig. 7:**
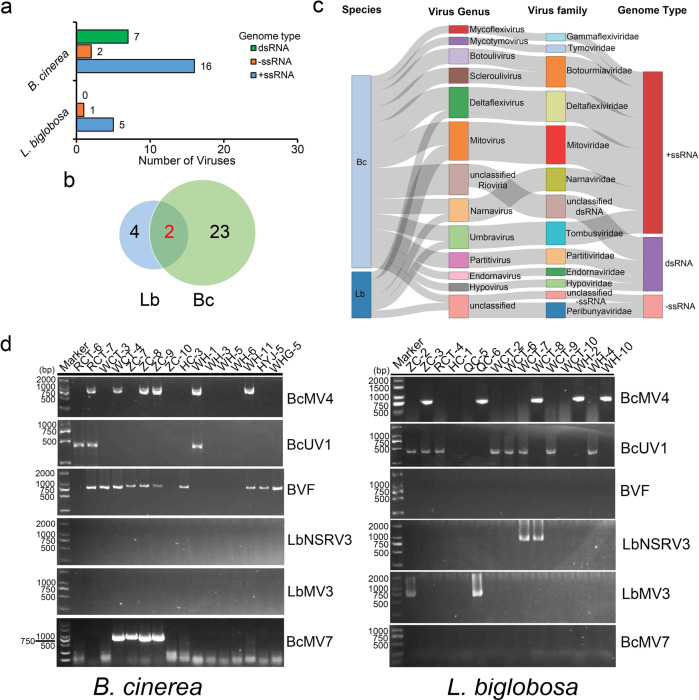
The diversity of mycoviruses detected by RNA-sequencing in *Leptosphaeria biglobosa* and *Botrytis cinerea* strains isolated from co-infection samples of *Brassica* crops. **a** The number of mycoviruses belonging to different genome types presents in each fungal population. **b** Venn diagram showed the number of mycoviruses present in *L. biglobosa* (Lb) and *B. cinerea* (Bc) populations. **c** Sankey diagram displaying the compositions of mycovirome from the populations of *L. biglobosa* and *B. cinerea*. **d** Detection of the presence of six mycoviruses in *L. biglobosa* and *B. cinerea* isolates by RT-PCR using the specific primers listed in Table S7. Note that two mycoviruses, Botrytis cinerea umbra-like virus 1 (BcUV1) and Botrytis cinerea mitovirus 4 (BcMV4), were detected in both two fungal populations.

Since co-infections of *Brassica* crops by both *B. cinerea* and *L. biglobosa* were observed under field conditions during disease surveys (Fig. 1), it is possible that viral cross-species transmission occurs between the two fungal pathogens. Total RNA of 15 *L. biglobosa* strains and 16 *B. cinerea* strains originated from coinfection samples were extracted and sequenced, and the presence of viruses in each fungal group was filtered out (Table S4). Moreover, the identities of all strains of *L*. *biglobosa* and *B*. *cinerea* were also confirmed by using PCR with the species-specific primers (Fig. S9). The results showed that *B. cinerea* strains have more potential viral species than *L. biglobosa* strains (Fig. 7a, b, c and Tables S5, 6). Combined with the results of PCR detection (Fig. S10), the 16 *B. cinerea* strains contained possibly 25 viral species, which could be assigned into 11 families (Table S6). By contrast, the 15 *L. biglobosa* strains contained possibly six viral species, which could be assigned into 4 families (Table S5). However, LbBV1 was not detected in either of the two fungal populations. Instead, 6 viral species were detected in both fungal pathogens with the high-throughput sequencing (Fig. 7d, Table S5, S6). Specific primers were designed based on these viral sequences (Table S7), and the presence of the two viruses was confirmed by using RT-PCR. The results showed that Botrytis cinerea umbra-like virus 1 (BcUV1) and Botrytis cinerea mitovirus 4 (BcMV4) were detected in both *B. cinerea* and *L. biglobosa* (Fig. 7d, Table S8).

## Discussion

The results of genomic structure, sequence homology, and phylogenetic analysis supported that LbBV1 was a member in the genus *Botybirnavirus*. Besides two dsRNA genome segments, LbBV1 also had spherical virons (~37 nm) with similar size (35–40 nm in diameter) to other botybirnaviruses [64]. Two major structural proteins (~100 and 90 kDa) were detected from the purified particles of LbBV1 by using SDS-PAGE analysis. The structural proteins of other botybirnaviruses, including ABV1 [65], have been determined to be in the N-proximal regions of two polypeptides encoded by the two dsRNAs. As LbBV1 showed high sequence homology to ABV1, we suppose that the estimated coding regions for two structural proteins were also in the N-proximal polypetides encoded by ORF1 and ORF2, respectively.

Mycoviral infection is generally thought to be benign due to their limited transmission capacity among different individuals. The belief is further enforced by the recent discovery of huge number of viral species in different fungal populations with the help of high-throughput sequencing technology [66–69]. In the present study, the infection of LbBV1 in the phytopathogenic fungus *L. biglobosa* was also asymptomatic, as all *L. biglobosa* strains with different genetic backgrounds carrying LbBV1 showed similar biological characteristics to those LbBV1-free strains of *L. biglobosa* (Fig. 3). However, LbBV1 could also infect *B*. *cinerea* and conferred hypovirulence to *B*. *cinerea*. Purified virons of LbBV1 were introduced into *B*. *cinerea* strain t-459. RT-PCR detection and northern blotting confirmed the infection of LbBV1 in *B*. *cinerea* strain t-459-V. LbBV1 infection reduced sclerotium production and virulence in *B*. *cinerea*, with minimum effect on mycelial growth. This indicated that viral cross-species transmission may also result in diseases of fungi, conferring hypovirulence to new phytopathogenic fungal hosts. Moreover, we found that the phenotypical changes mediated by LbBV1 in *B*. *cinerea* were not so severe, as the growth of strain t-459-V was almost normal and its pathogenicity was not fully lost. All these relatively mild changes may help ensure the survival of LbBV1-infected *B*. *cinerea*.

Cross-species transmission of LbBV1 between *L. biglobosa* and *B*. *cinerea* was also demonstrated by co-culturing conidia of the two fungi on PDA and co-inoculation on oilseed rape stem at the transmission rate of about 4.6% and 18.8%, respectively. Although the transmission of mycoviruses was believed to be limited, the occurrence of mycoviruses in different fungal hosts indicates that cross-species transmission may occur in nature [60, 70]. Moreover, cross-species transmission of mycoviruses has also been reported in other fungi under laboratory conditions, like *Aspergillus niger*/*A*. *nidulans* [71], *S*. *sclerotiorum*/*S*. *minor* [72], and *C*. *parasitica*/*C*. *nitschkei* [73]. In all these cases, the donor and recipient species belonged to the same genus, and the transmission was accomplished by using the pairing culture technique. In the present study, successful transmission was demonstrated only when conidia of the two fungal species were co-cultured, a condition that may also occur in nature. Therefore, mycoviral transmission may occur more frequently at the initial stage of infection for two or more fungi co-infecting same plant tissues than through pairing culture under laboratory conditions. Possible reasons include at the initial stage of spore germination and germ tube growth the vegetative incompatible reaction may not be so strong, offering more chances to contact each other in mixed spores relative to the pairing culture technique.

We believe that cross-species transmission of LbBV1 may have occurred in nature several times. The high sequence identity between LbBV1 in *L. biglobosa* and ABV1 in *Alternaria alternata* indicates a possible direct or indirect cross-species transmission between the two distantly related species. *Leptosphaeria* and *Alternaria* belong to different families, *Leptosphaeria* and *Pleosporaceae*, respectively. Besides being close related to ABV1, LbBV1 also showed close relationship to IaBV1 (Ipomoea aquatica botybirnavirus 1) and BcBV1 (Fig. 2c). The genus *Botrytis* is in the class *Leotiomycetes*, order *Heliotiales* and family *Sclerotiniaceae*, which is farther away than *Alternaria* to the genus *Leptosphaeria*. Such a close sequence similarity suggests that a cross-species transmission of the ancestor of LbBV1 may have occurred between *Botrytis* and *Leptosphaeria* rather than the co-evolution of the LbBV1 ancestor along with their hosts during speciation of the two fungi. Besides the case of cross-species transmission of LbBV1, some other botybirnaviruses have also been detected in different fungal hosts, indicating the possible cross-species transmission of botybirnaviruses in nature. For example, BpBV1 has been detected in both *B*. *squamosa* [74] and *S*. *sclerotiorum* [75] in addition to its earliest recorded host—*B*. *porri*.

As co-infections of oilseed rape by both *B*. *cinerea* and *L*. *biglobosa* have been observed in the field, we hypothesize that the transmission of LbBV1 from *L*. *biglobosa* to *B*. *cinerea* may also occur during the co-infection (Fig. 1). Furthermore, we also want to test whether other viruses may be transmitted from one species to the other during co-infection. To test this hypothesis, diseased samples of *Brassica* crops showing symptoms of co-infection by both pathogens were collected, and the *B*. *cinerea* and *L*. *biglobosa* strains were subsequently isolated and purified. Total RNAs were extracted from *B*. *cinerea* and *L*. *biglobosa* strains, and then used in RNA-seq. As LbBV1 was not detected in either of the two fungal populations, we also tried to collect and analyze samples from where *L*. *biglobosa* strain GZJS19 was originally collected, but no sample showing co-infection was obtained. However, two other mycoviruses, BcMV4, and BcUV1, were detected in both fungal species. This indicates that viral cross-species transmission may occur if two fungi share same or similar ecological niche. In this specific case, there is a question that which fungus is the original host of these two viruses. To address this question, the sequences of two viruses were searched in NCBI database, and all of them marched viruses in *B*. *cinerea* [69, 76]. Moreover, the two viruses were also present in the viral-like contigs identified in the RNA-seq data from Chinese *B*. *cinerea* populations containing 500 strains [77]. In addition, the two viruses were not found in the viral-like contigs from the RNA-seq data from Chinese *L*. *biglobosa* populations containing 400 strains (unpublished data). Therefore, the evidence suggests that the original host of the two viruses is *B*. *cinerea* but not *L*. *biglobosa*, and the presence of the two viruses in *L*. *biglobosa* strains is likely due to the viral cross-species transmission from *B. cinerea* to *L. biglobosa*. We detected six possible mycoviral species in both fungal groups in RNA-seq, but only two of them were detected in the two fungi by using RT-PCR. A possible explanation is that viral cross transmission might occur for all six viruses, but adapting new hosts may not be successful, and the other four viruses may vanish during the subculture process.

Although cross-species transmission of mycoviruses was reported as mentioned above, the effects of viral interspecies transmission on fungal biology in most cases remain unclear. However, some other circumstantial evidence suggest that interspecies transmission of mycoviruses may be accompanied with phenotypical changes in fungal hosts. For example, same species of mycoviruses were reported in different fungal hosts, like Botrytis cinerea mitovirus 1 (BcMV1) [60, 63], Ophiostoma novo-ulmi mitovirus 3a (OnMV3a) [70], Botrytis cinerea mymonavirus 1 [59], and BpBV1 [62], suggests that cross-species transmission may occur among different fungi even if the two fungi are evolutionarily distantly related to each other. It is notable that virus-mediated phenotypical changes vary greatly for the same virus in different fungal hosts. For example, BcMV1 and OnMV3a are associated with the hypovirulence of *B*. *cinerea* and *S*. *homoeocarpa*, respectively, but no significant effect on *O*. *novo-ulmi* was observed for the two viruses [60, 63, 70]. In addition, some in-lab evidence also indicates that phenotypic alterations induced by same mycovirus may vary between different hosts [78, 79]. Different from mycoviruses, many solid lines of evidence confirm that cross-species transmission in animal viruses is often able to result in diseases of humans and animals, sometimes even human pandemics, such as influenza A virus [80, 81], human immunodeficiency virus-1 [82], and severe acute respiratory syndrome coronavirus-2—the causal agent of current pandemics [53]. Similarly, recent high-throughput sequencing studies revealed that diverse viruses persist in wild plants, and they are generally asymptomatic in wild plants [83], and are believed to be reservoirs of crop viruses, which may cause diseases on infected crops [84, 85]. Therefore, it is worthy to test whether similar cases also occur in fungi.

In the present study, we found that similar situation may also exist in mycoviruses. Mycoviral transmission can be accomplished between two fungal species distantly related to each other, and viruses having no apparent effects on one fungal host may cause significant phenotypical changes on the other one after interspecies transmission. This may happen under natural conditions if even two fungi share same or similar ecological niches, and the related clue can be also found in the literature. For example, the presence of Trichoderma koningiopsis totivirus 1 in *Trichoderma koningiopsis* strain Mg10 and *Clonostachys rosea* strain Mg06 isolated from the same soil sample suggest the viral interspecies transmission between the two fungi [86]. Moreover, some observations also implied that mycoviruses can be acquired during fungal vegetative growth under natural conditions without the limitation of vegetative incompatibility [87, 88]. In the present study, cross-species transmission of LbBV1 from *L*. *biglobosa* to *B*. *cinerea* was not successful by using pair-culture technique, but succeeded when co-inoculation of the conidia of the two fungi on PDA and on plants. This indicates that mycoviral interspecies transmission may occur under some circumstances beyond the familiar ways, as natural conditions are much more complex than in the laboratory. For instance, one recent case showed that plant viruses may assist the transmission of hypoviruses between different fungal species [89]. As mentioned above, due to the limited transmissibility of mycoviruses, hypovirulence mediated by mycoviruses may be detrimental for both virus and host fungus, as they may not be able to survive in the long term. Therefore, most mycoviral infections are benign. However, hypovirulence-associated mycoviruses have been widely reported in many phytopathogenic fungi, which is contrary to their benign infection, and most of their host fungal strains were initially isolated from diseased plants showing typical symptoms. Therefore, the present study provides an explanation for why hypovirulence-associated mycoviruses were widely reported in many phytopathogenic fungi, as these viruses might invade their hosts recently. These results also support the hypothesis that hypovirulence traits mediated by viruses may be a consequence of viral invasions in new fungal hosts by transmission between species [38].

**Fig. 8:**
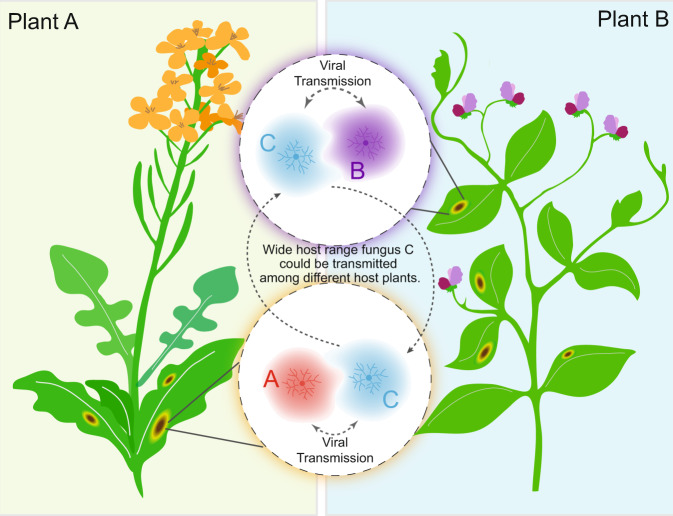
A possible explanation of the cross species transmission between two phytopathogenic fungi, fungus A and B, sharing no similar ecological niches. Fungus C has a wide host range and can infect both plants A and B. During the infection process, fungus C may infect plant A along with fungus A, the host-specific pathogen of plant A, at the same infection site. Thus, viruses may be able to be transmitted between A and C, no matter from A to C, or the opposite. Similarly, this case may also occur between fungus B and C, of which fungus B is the host-specific pathogen of plant B. As fungus C can be transmitted between plants A and B, it may play as a link or bridge helping the viral transmission between fungus A and B.

Cross-species transmission in fungi may be different from that in animals. In animal viruses, a few animals are considered as reservoirs of viruses, such as bats [45]. In contrast, we suppose that many fungi can serve as reservoirs to each other, as one fungal species may harbor numerous viruses as shown in recent high through-put sequencing analysis [66–69]. In the present study, LbBV1 could be transmitted from *L*. *biglobosa* to *B*. *cinerea*. In addition, the two viruses (BcMV4 and BcUV1) were also suggested to be transmitted from *B*. *cinerea* to *L*. *biglobosa*. Thus, both *L*. *biglobosa* and *B*. *cinerea* each can serve as reservoirs to the other. However, viral diversity in different fungal species vary greatly. In this study, *B*. *cinerea* contained more virus species than did *L*. *biglobosa*, although both fungal groups had almost same number of strains and were isolated from same geographic locations. Based on these observations, we speculate that phytopathogenic fungi with wide host range may tend to contain more species of viruses, as they should have more chances to contact with more diverse fungi and may have a wider geographic distribution. In addition, these phytopathogenic fungi, like *B*. *cinerea* and *S*. *sclerotiorum*, could also play as a bridge or link for viruses among different fungal species (Fig. 8). This may be helpful to explain why two fungi in different ecological niches can still carry the same virus. For example, *O*. *novo-ulmi* is a fungal pathogen causing Dutch elm disease on elm trees, whereas *S*. *homoeocarpa* is the causal agent of dollar spot on turfgrasses. Although they are in different ecological niches, the same virus—Ophiostoma novo-ulmi mitovirus 3a (OnMV3a) could be detected in both fungi [70]. In this case, one fungus living on both elm tree and turfgrass might be able to transmit OnMV3a between *O*. *novo-ulmi* and *S*. *homoeocarpa*. Therefore, we believe that further studies on fungal groups of saprophytes, epiphytes, and endophytes should be explored, as all of them have lots of chances to contact with other phytopathogenic fungi.

For a long time, investigations of mycoviruses have mostly focused on viral groups that manifest significant biological effects, especially hypovirulence-associated viruses in phytopathogenic fungi, as one important goal of mycoviral research is to explore viral resources for biocontrol of plant fungal disease. In contrast, mycoviruses that have no significant biological effects have received much less attention, and many of which just have sequence information. However, the present study provides a new insight for mycoviruses mostly seen to be asymptomatic. They may have no significant effects on one fungal host, but their host shifts may lead to great phenotypical changes on new fungal hosts, which may have the potential for further development as biocontrol agents against these new hosts.

## Supplementary information

Supplementary tables and figures

## Data Availability

The Genbank accession numbers of dsRNA 1 and dsRNA 2 of LbBV1 are MZ612795 and MZ612796, respectively. The raw sequencing reads reported in the present study have been deposited in the Sequence Read Archive (SRA) database: BioProject accession no. PRJNA778037, BioSample accession no. SAMN22899792 (*L*. *biglobosa*) and SAMN22899788 (*B*. *cinerea*), and SRA accession no. SRS10959936 (*L*. *biglobosa*) and SRS10959934 (*B*. *cinerea*).
