# Supplementary material for: Viral cross-class transmission results in disease of a phytopathogenic fungus
Source: ISME J. 2022 Aug 31;16(12):2763–74. doi: 10.1038/s41396-022-01310-y (PMC9428384; doi:10.1038/s41396-022-01310-y)
Supplement: Supplementary file 1 — Supplementary tables and figures [file 41396_2022_1310_MOESM1_ESM.pdf]

# Supplementary information

# Viral Cross-class Transmission Results in Disease of a Phytopathogenic Fungus

Yue Deng[1,2], Kang Zhou[1,2], Mingde Wu[1,2*], Jing Zhang[1,2], Long Yang[1,2], Weidong Chen[3], Guoqing Li[1,2]

[1] State Key Laboratory of Agricultural Microbiology, Huazhong Agricultural University, Wuhan 430070, China

[2] Hubei Key Laboratory of Plant Pathology, Huazhong Agricultural University, Wuhan 430070, China

[3] U.S. Department of Agriculture, Agricultural Research Service, Washington State University, Pullman, WA 99164, USA

[*]Corresponding author: Dr. Mingde Wu, E-mail: mingde@mail.hzau.edu.cn

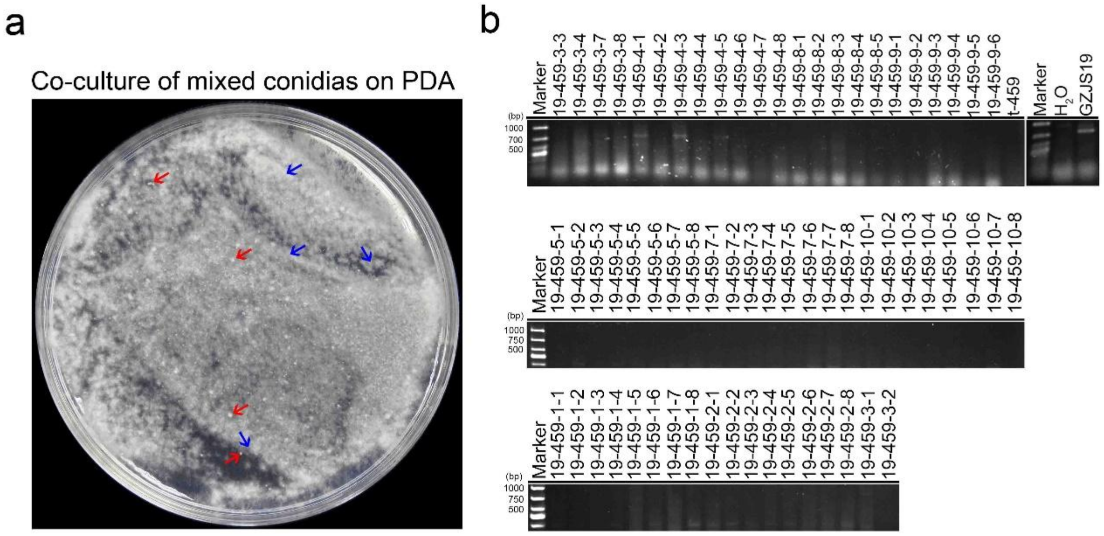


**Supplementary Figure S1. Cross species transmission of LbBV1 from *Leptosphaeria biglobosa* strain GZJS19 to *Botrytis cinerea* strain t-459 by co-culturing the conidia of two fungi on PDA plate. a**, Co-culture morphology of conidia of *B. cinerea* strain t-459 and *L. biglobosa* strain GZJS19 on PDA plate. Red and blue arrowheads indicate the possible presence of mycelia of *L. biglobosa* and *B. cinerea*, respectively. **b**, RT-PCR detection of LbBV1 in derivative *B. cinerea* strains obtained from strain t-495 after co-culturing with *L. biglobosa* strain GZJS19.

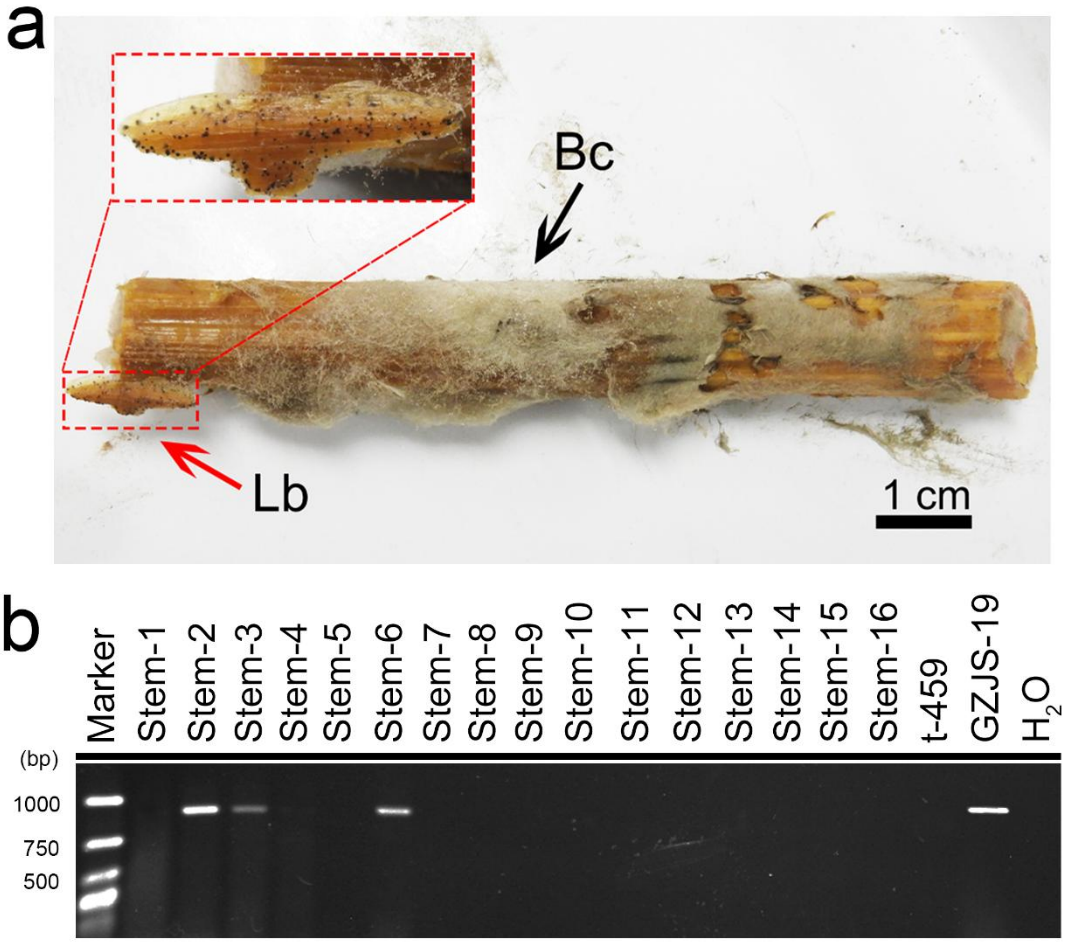


**Supplementary Figure S2. Cross species transmission of LbBV1 from *Leptosphaeria biglobosa* strain GZJS19 to *Botrytis cinerea* strain t-459 by co-inoculating the conidia of two fungi on the stem of oilseed rape. a**, Co-inoculation of conidia of *B. cinerea* strain t-459 and *L. biglobosa* strain GZJS19 on an oilseed rape stem. Note the formation of conidia and mycelium (black arrowhead) of *B. cinerea* and pycnidia (red arrowhead and enlarged in dashed line box) of *L. biglobosa* on the stem. **b**, RT-PCR detection of LbBV1 in derivative *B. cinerea* strains re-isolated from the oilseed rape stem co-inoculated with *B. cinerea* strain t-459 and *L. biglobosa* strain GZJS19.

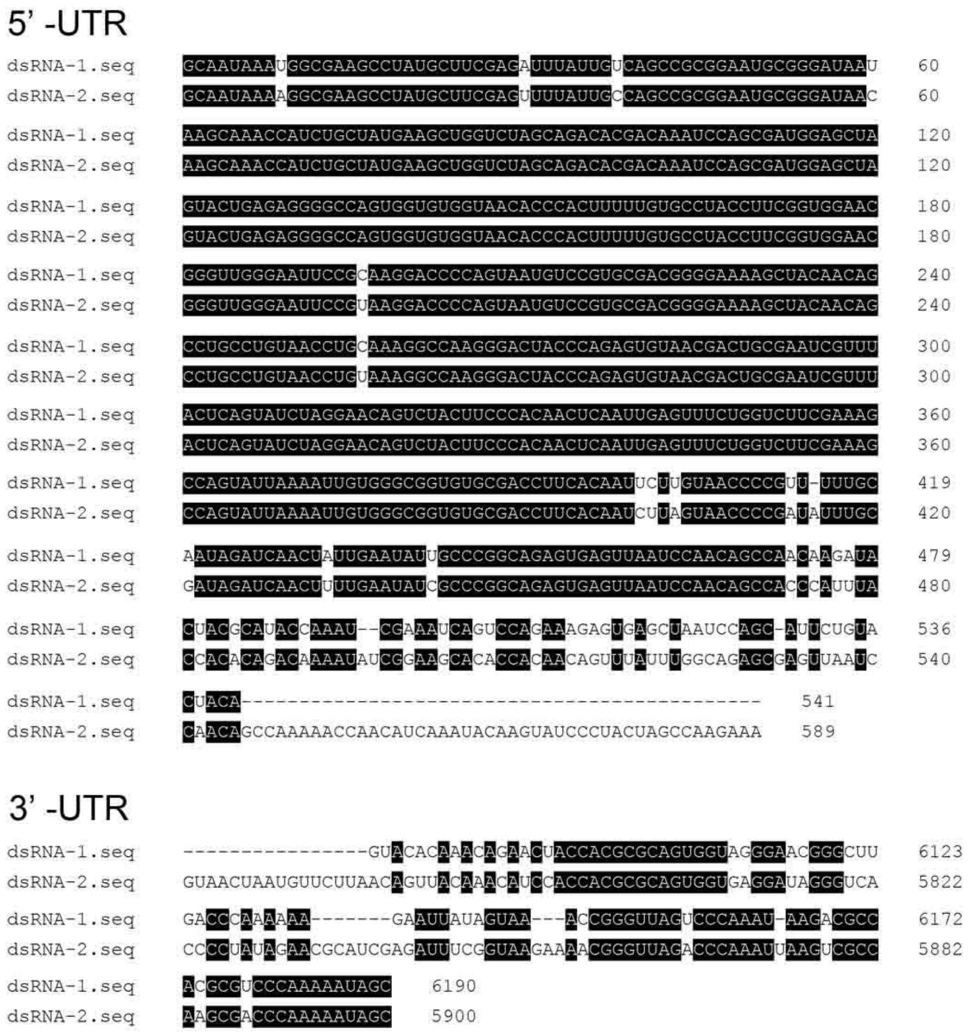


**Supplementary Figure S3. Alignment of 5'- UTR and 3'-UTR sequences of the coding strands of dsRNA-1 and dsRNA-2 of LbBV1.**

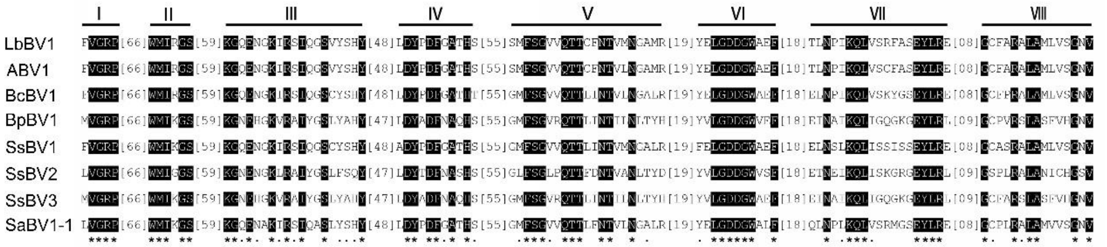


**Supplementary Figure S4. Multiple alignment of RNA-dependent RNA polymerase (RdRp) sequences of Leptosphaeria biglobosa botybirnavirus 1 (LbBV1) and other RNA viruses showing the structures of the eight motifs (I to VIII).**

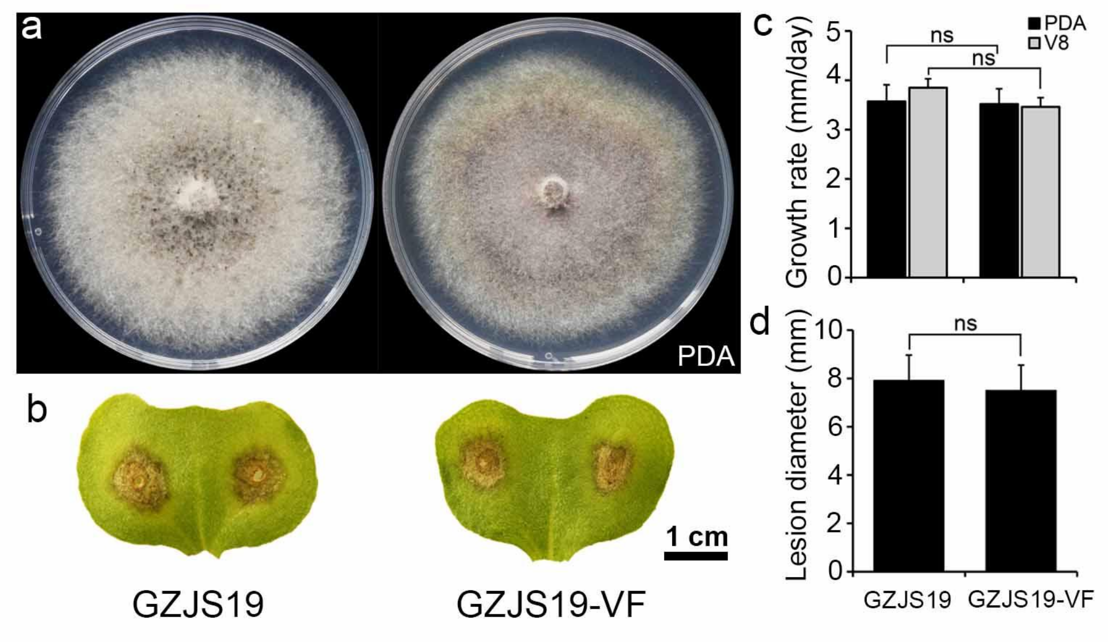


**Supplementary Figure S5. Biological properties of *Leptosphaeria biglobosa* strains GZJS19 (LbBV1 infected) and GZJS19-VF (LbBV1 free). a**, Colony morphology (23°C, 10 days) of strains GZJS19 and GZJS19-VF on potato dextrose agar (PDA). **b**, Pathogenicity assay (23°C, 7 days) of strains GZJS19 and GZJS19-VF on cotyledons of oilseed rape. **c**, Radial mycelial growth rate (23°C) of strains GZJS19 and GZJS19-VF on PDA and V8. **d**, Lesion diameter of strains GZJS19 and GZJS19-VF (23°C, 7days) on cotyledons of oilseed rape. The results are expressed as arithmetic means the standard errors of the means, "ns" means no significant difference ($p > 0.05$) between strains GZJS19 and GZJS19-VF in mycelial growth rate and virulence.

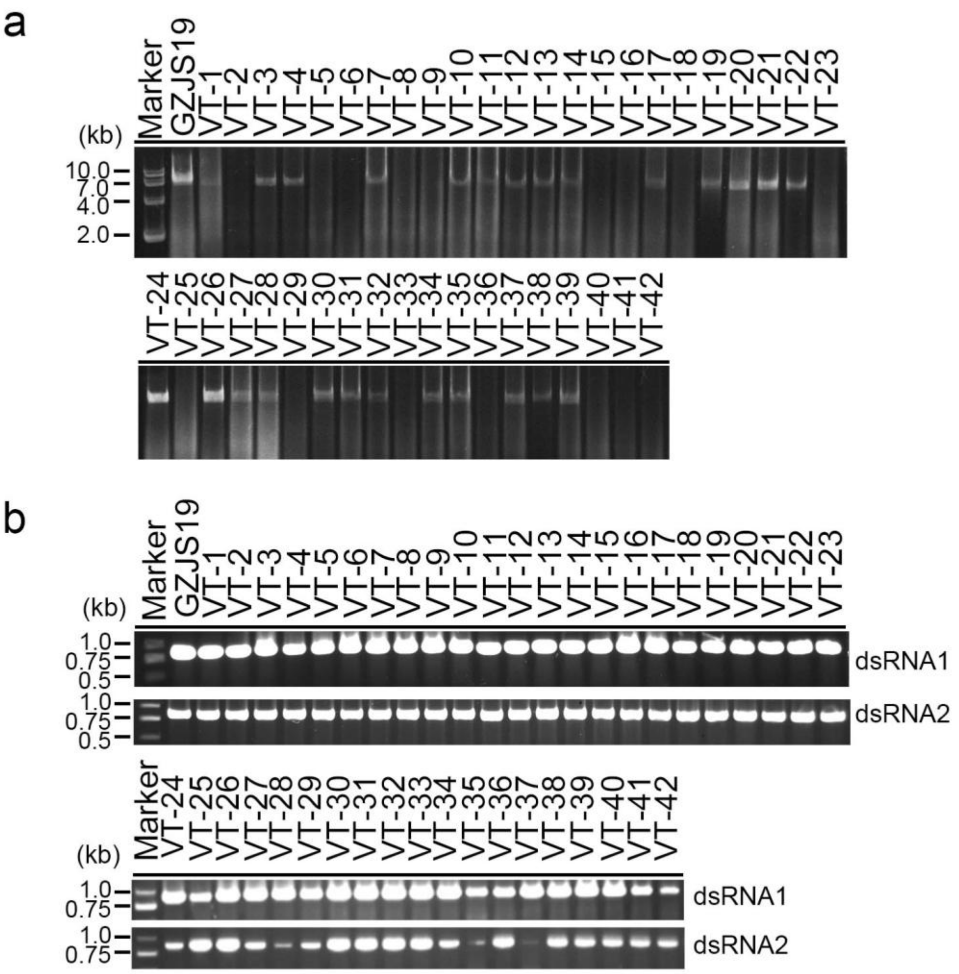


**Supplementary Figure S6. Detection of LbBV1 dsRNA in single conidium strains derived from *Leptosphaeria biglobosa* strain GZJS19. a**, Detection of LbBV1 dsRNA in single conidium strains derived from *L. biglobosa* strain GZJS19. **b**, Detection of LbBV1 in single conidium strains derived from *L. biglobosa* strain GZJS19 by RT-PCR. Note that although some strains showed no presence of LbBV1 dsRNA, RT-PCR detection showed these strains were infected by LbBV1.

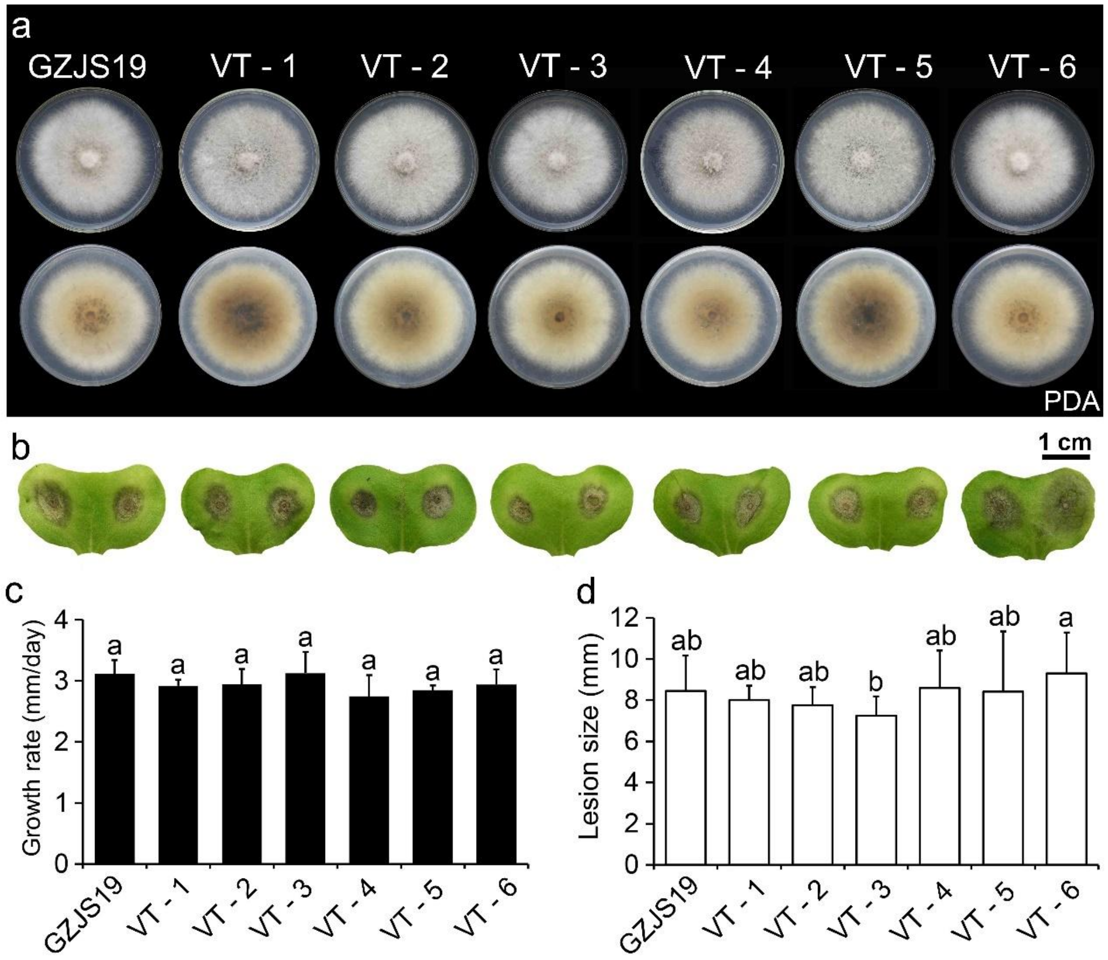


**Supplementary Figure S7. Biological properties of six single conidium progenies of *Leptosphaeria biglobosa* strain GZJS19. a**, Colony morphology (23°C, 10 days) of strains GZJS19 and its six single conidium progenies on potato dextrose agar (PDA). **b**, Pathogenicity assay (23°C,7 days) of strains GZJS19, VT-1, VT-2, VT-3, VT-4, VT-5, VT-6 and Lb731 on cotyledons of oilseed rape. **c**, Radial mycelial growth rate (23°C) of strains GZJS19 and its six single conidium progenies on PDA. **d**, Lesion diameter of strains GZJS19 and its six single conidium progenies (23°C, 7days) on cotyledons of oilseed rape. The results are expressed as arithmetic means the standard errors of the means. In each histogram, bars labeled with the same letters are not significantly different ($p > 0.05$) according to the least-significant-difference test.

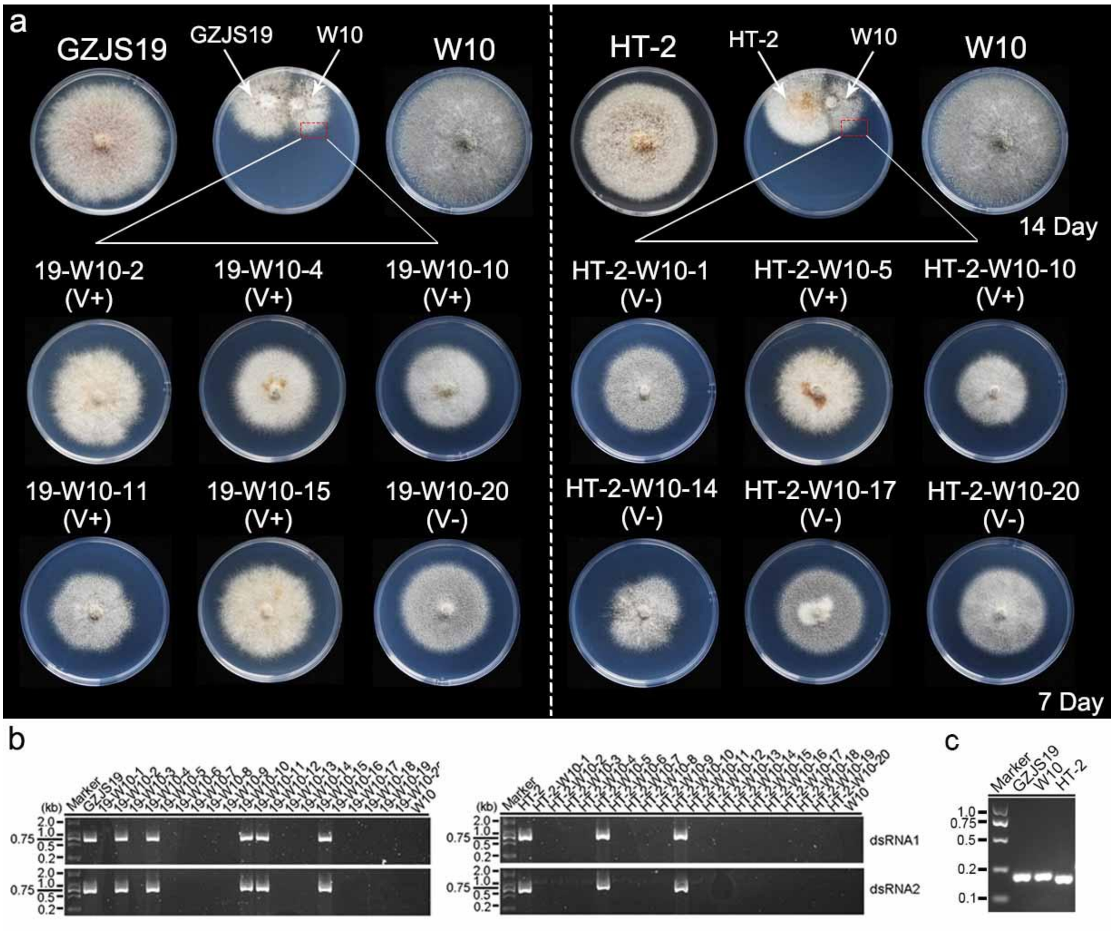


**Supplementary Figure S8. Horizontal transmission of LbBV1 from *Leptosphaeria biglobosa* strains GZJS19 and HT-2 to strain W10 by using pairing culture technique. a**, Colony morphology (23°C, 7 days) of strains GZJS19, W10, HT-2 and their derivative strains obtained from strain W10 after pair-culturing with strain GZJS19 and HT-2, respectively. **b**, RT-PCR detection of the presence of dsRNA-1/dsRNA-2 in all derivative strains by using specific primers. **c**, Simple sequence repeats (SSR) analysis of strains GZJS19, HT-2 and W10 by using agarose gel electrophoresis.

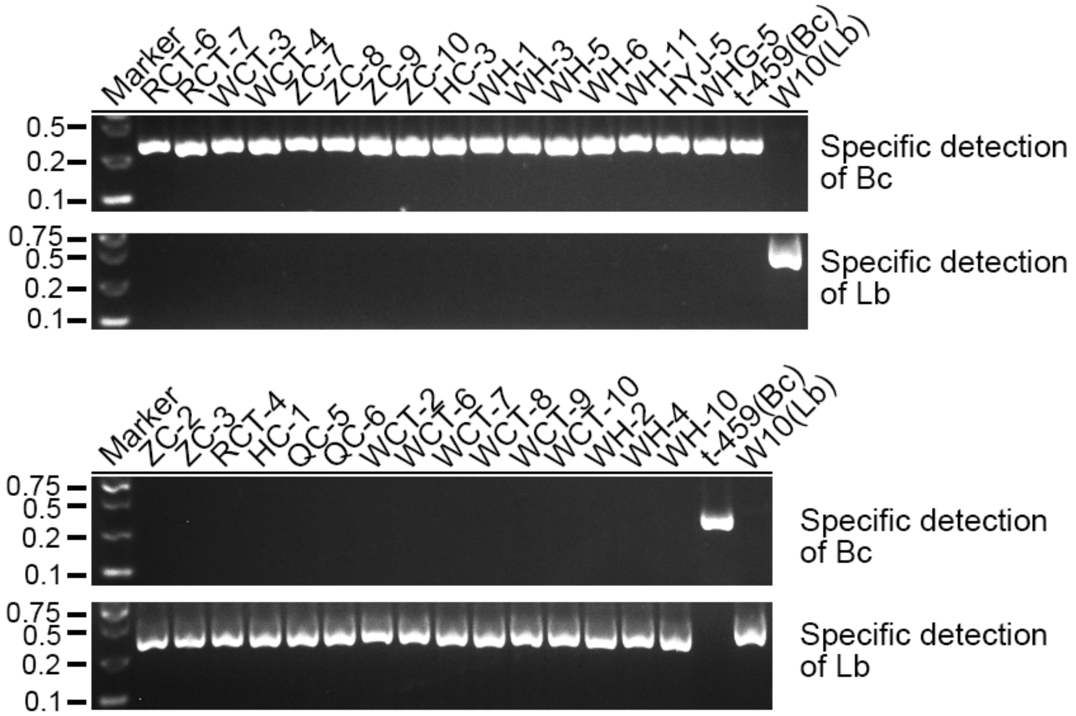


**Supplementary Figure S9. Specific identification of *Leptosphaeria biglobosa* and *Botrytis cinerea* strains isolated from co-infection samples of *Brassica* crops.** Specific identification of two fungal species strains was confirmed by PCR using the species specific primers listed in Table S2, Bc = *B. cinerea*, Lb = *L. biglobosa*.

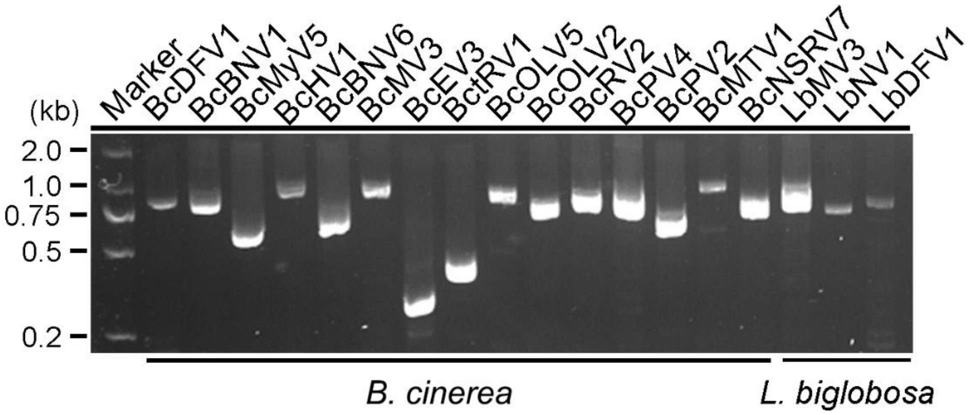


**Supplementary Figure S10. RT-PCR detection of mycoviral contigs in *Leptosphaeria biglobosa* and *Botrytis cinerea* samples.** The primers used for RT-PCR were designed according to the sequences of putative mycoviral contigs, and the information of primers was listed in Table S7.

Supplementary Table S1. Original strains of *Leptosphaeria biglobosa* and *Botrytis cinerea* used for biological property tests.

| Strain | Host | Province | Year | Ref. etc. |
|---|---|---|---|---|
| GZJS19 | *Brassica campestris* | Guizhou | 2017 | In this study |
| Lb731 | *Brassica napus* | Henan | 2017 | In this study |
| Lb681 | *Brassica napus* | Yunnan | 2017 | In this study |
| Lb1176 | *Brassica napus* | Qinghai | 2017 | In this study |
| Lb1168 | *Brassica napus* | Qinghai | 2017 | In this study |
| W10 | *Brassica napus* | Hubei | 2013 | Hygromycin resistant |
| HBtom-459 (t-459) | *Solanum lycopersicum* | Hubei | 2014 | Hao et al.2018[1] |

[1] Hao F, Ding T, Wu MD, Zhang J, Yang L, Chen W, Li GQ. Two novel hypovirulence-associated mycoviruses in the phytopathogenic fungus *Botrytis cinerea*: Molecular characterization and suppression of infection cushion formation. *Viruses*. 2018;10, 254.

Supplementary Table S2. Oligonucleotide primers used for strain specific identification in this study.

| Primer name | Sequence (5'→3') | Size | Reference |
|---|---|---|---|
| SSR17-F | GACGACTGCACGACAACATC | 178-199 | unpublished |
| SSR17-R | ACTCGCCTACCAACATGGAC | | |
| LbigF | ATCAGGGGATTGGTGTCAGCAGTTGA | 441 | Liu et al. 2006[1] |
| LmacR | GCAAAATGTGCTGCGCTCCAGG | | |
| Bc-F | CAGGAAACACTTTTGGGGATA | 327 | Fan et al. 2015[2] |
| Bc-R | GAGGGACAAGAAAATCGACTAA | | |

[1] Liu S Y, Liu Z, Fitt B D L, Evans N, Foster SJ, Huang YJ, Latunde-Dada AO and Lucas JA. Resistance to *Leptosphaeria maculans* (phoma stem canker) in *Brassica napus* (oilseed rape) induced by *L. biglobosa* and chemical defence activators in field and controlled environments. *Plant Pathol*. 2006;55:401–412.

[2] Fan X, Zhang J, Yang L, Wu MD, Chen WD and Li GQ. Development of PCR-based assays for detecting and differentiating three species of *Botrytis* infecting broad bean. *Plant Dis*. 2015;99(5):691-698.

Supplementary Table S3. Oligonucleotide primers/adaptor used for cDNA cloning and revere transcription (RT)-PCR detection of Leptosphaeria biglobosa botybirnavirus 1

| Primer name | Sequence(5’→3’) | Position | Polarity |
|---|---|---|---|
| 1-1g-F | GGCTAGAGAACGACAGCGTA | 5737-5756 | + |
| 1-1g-R | CGGACACCGCATCTTCCATC | 3709-3729 | - |
| 1-2g-F | TGAAGTGGAAGGCGATGGAAG | 3701-3721 | + |
| 1-2g-R | CAGCTCGACAACAAGGTCGG | 894-913 | - |
| 1-5t-R | AATGTCTTCGGGGACGGTAG | 5890-5910 | - |
| 1-3t-F | GAGCTGAAGCAGCGTATTGC | 704-723 | + |
| 2-1g-F | TTGTACCTCATGGGCAACGG | 1635-1654 | + |
| 2-1g-R | ATCACGCTTTGCTTGCCTTC | 3214-3233 | - |
| 2-2g-F | ACTGAAGACACCACACCACC | 3120-3139 | + |
| 2-2g-R | TGTACTCTCCACATCCCGCT | 4831-4850 | - |
| 2-5t-R | GCCAGCTTCGTATTGAAGGC | 984-1003 | - |
| 2-3t-F | ACCTCGCCAAAGCACATACA | 4090-4109 | + |
| RC110A | GGGGGACACGCCGATAAGATA | complementary to the adaptor 110A | +/- |
| 110A | TATCTTATCGGCGTGTCCCCC | to 3’-end of dsRNA | +/- |
| RT-1-F | GCTGCCTATTGGAGTTGAGGA | 1063-1083 | + |
| RT-1-R | TGGCTTGGTCACGGTCTTG | 1915-1933 | - |
| RT-2-F | AGCCGATTTTCCGCAGTTTG | 1016-1035 | + |
| RT-2-R | CGTGCTATGGCCATCTGAGT | 1866-1885 | - |
| Probe1-F | TGCGATCAGTTCGATCCAGG | 1228-1247 | + |
| Probe1-R | CTTTTGCCTCTCCCGCATTC | 1678-1697 | - |
| Probe2-F | TTAGGACCAAACGAGTCCAGC | 2318-2337 | + |
| Probe2-R | AGTGCGTAGCTCCGAAATCTG | 2958-2977 | - |

Supplementary Table S4. *Leptosphaeria biglobosa* and *Botrytis cinerea* strains isolated from co-infection samples of used for RNA sequencing in this study.

| Sample name | Strain | Host | Year | Location | Country | Fungal species |
|---|---|---|---|---|---|---|
| Lb-Bcp-1 | RCT-4 | *Brassica compestris* var. *purpurea* | 2019 | Wuhan | China | *Leptosphaeria biglobosa* |
| Lb-Bcu-1 | WCT-2 | *Brassica campestris* var. *utilis* | 2019 | Wuhan | China | *Leptosphaeria biglobosa* |
| Lb-Bcu-2 | WCT-6 | *Brassica campestris* var. *utilis* | 2019 | Wuhan | China | *Leptosphaeria biglobosa* |
| Lb-Bcu-3 | WCT-7 | *Brassica campestris* var. *utilis* | 2019 | Wuhan | China | *Leptosphaeria biglobosa* |
| Lb-Bcu-4 | WCT-8 | *Brassica campestris* var. *utilis* | 2019 | Wuhan | China | *Leptosphaeria biglobosa* |
| Lb-Bcu-5 | WCT-9 | *Brassica campestris* var. *utilis* | 2019 | Wuhan | China | *Leptosphaeria biglobosa* |
| Lb-Bcu-6 | WCT-10 | *Brassica campestris* var. *utilis* | 2019 | Wuhan | China | *Leptosphaeria biglobosa* |
| Lb-Bcc-1 | QC-5 | *Brassica campestris* var. *chinensis* | 2019 | Wuhan | China | *Leptosphaeria biglobosa* |
| Lb-Bcc-2 | QC-6 | *Brassica campestris* var. *chinensis* | 2019 | Wuhan | China | *Leptosphaeria biglobosa* |
| Lb-Bjt-1 | ZC-2 | *Brassica juncea* var. *tumida* | 2019 | Wuhan | China | *Leptosphaeria biglobosa* |
| Lb-Bjt-2 | ZC-3 | *Brassica juncea* var. *tumida* | 2019 | Wuhan | China | *Leptosphaeria biglobosa* |
| Lb-Bob-1 | HC-1 | *Brassica oleracea* var. *botrytis* | 2019 | Wuhan | China | *Leptosphaeria biglobosa* |
| Lb-Bn-1 | WH-2 | *Brassica napus* | 2019 | Wuhan | China | *Leptosphaeria biglobosa* |
| Lb-Bn-2 | WH-4 | *Brassica napus* | 2019 | Wuhan | China | *Leptosphaeria biglobosa* |
| Lb-Bn-6 | WH-10 | *Brassica napus* | 2019 | Wuhan | China | *Leptosphaeria biglobosa* |
| Bc-Bcp-1 | RCT-6 | *Brassica compestris* var. *purpurea* | 2019 | Wuhan | China | *Botrytis cinerea* |
| Bc-Bcp-2 | RCT-7 | *Brassica compestris* var. *purpurea* | 2019 | Wuhan | China | *Botrytis cinerea* |
| Bc-Bcu-3 | WCT-3 | *Brassica campestris* var. *utilis* | 2019 | Wuhan | China | *Botrytis cinerea* |
| Bc-Bcu-8 | WCT-4 | *Brassica campestris* var. *utilis* | 2019 | Wuhan | China | *Botrytis cinerea* |
| Bc-Bjt-2 | ZC-7 | *Brassica juncea* var. *tumida* | 2019 | Wuhan | China | *Botrytis* |

| | | | | | | |
|---|---|---|---|---|---|---|
| | | | | | | *cinerea* |
| Bc-Bjt-3 | ZC-8 | *Brassica juncea* var. *tumida* | 2019 | Wuhan | China | *Botrytis cinerea* |
| Bc-Bjt-4 | ZC-9 | *Brassica juncea* var. *tumida* | 2019 | Wuhan | China | *Botrytis cinerea* |
| Bc-Bjt-5 | ZC-10 | *Brassica juncea* var. *tumida* | 2019 | Wuhan | China | *Botrytis cinerea* |
| Bc-Bob-1 | HC-3 | *Brassica oleracea* var. *botrytis* | 2019 | Wuhan | China | *Botrytis cinerea* |
| Bc-Bn-1 | WH-1 | *Brassica napus* | 2019 | Wuhan | China | *Botrytis cinerea* |
| Bc-Bn-3 | WH-3 | *Brassica napus* | 2019 | Wuhan | China | *Botrytis cinerea* |
| Bc-Bn-5 | WH-5 | *Brassica napus* | 2019 | Wuhan | China | *Botrytis cinerea* |
| Bc-Bn-6 | WH-6 | *Brassica napus* | 2019 | Wuhan | China | *Botrytis cinerea* |
| Bc-Bn-11 | WH-11 | *Brassica napus* | 2019 | Wuhan | China | *Botrytis cinerea* |
| Bc-Bjc-1 | HYJ-5 | *Brassica juncea* var. *foliosa* | 2019 | Wuhan | China | *Botrytis cinerea* |
| Bc-Boa-1 | WHG-5 | *Brassica oleracea* var. *albiflora* | 2019 | Wuhan | China | *Botrytis cinerea* |

Supplementary Table S5. Assembled sequences with similarity to previously described viruses in *Leptosphaeria biglobosa* from the co-infection samples

| Number | Contig number | Number of reads | Contig length | GenBank accesion numbers | Name of putative viruses | Best match | aa identity (%) | Genome type | Family/Genus |
|---|---|---|---|---|---|---|---|---|---|
| 1 | Contig 9 | 2303 | 2442 | OL440113 | Leptosphaeria biglobosa mitovirus 3 (LbMV3) | AAO21337.1 RdRp-like protein [Sclerotinia homoeocarpa mitovirus] | 49.5 | +ssRNA | *Mitoviridae* |
| 2 | Contig 54 | 266 | 1312 | OL602077 | Leptosphaeria biglobosa negative ssRNA virus 3 (LbNSRV3) | QEQ12679.1 nucleocapsid protein [Penicillium roseopurpureum negative ssRNA virus 1] | 64.4 | -ssRNA | *Unclassfied* |
| 3 | Contig 50 | 19157 | 1346 | OL602078 | Leptosphaeria biglobosa deltaflexivirus 1 (LbDFV1) | YP_009508364.1 hypothetical protein [Sclerotinia sclerotiorum deltaflexivirus 1] | 58.8 | +ssRNA | *Deltaflexiviridae* |
| 4 | Contig 80 | 95 | 1113 | OL602079 | Leptosphaeria biglobosa narnavirus 1 (LbNV1) | QJV68034.1 putative RNA-dependent RNA polymerase [Magnaporthe oryzae narnavirus 1] | 79.0 | +ssRNA | *Narnaviridae* |
| 5 | Contig 2484 | 18 | 395 | OL602080 | Botrytis cinerea umbra-like virus 1 (BcUV1) | QJT73736 RNA-dependent RNA polymerase [Botrytis cinerea umbra-like virus 1] | 90.6 | +ssRNA | *Tombusviridae* |
| | Contig 34462 | 6 | 231 | - | Botrytis cinerea umbra-like virus 1 (BcUV1) | Botrytis cinerea umbra-like virus 1 | 93.2 | +ssRNA | *Tombusviridae* |
| 6 | Contig 38597 | 3 | 225 | OL602083 | Botrytis cinerea mitovirus 4 (BcMV4) | YP_009182163.1 RNA dependent RNA polymerase [Botrytis cinerea mitovirus 4] | 94.4 | +ssRNA | *Mitoviridae* |

Note: BcUV1 contained two contigs in *L. biglobosa* samples. The grey shading filled areas in the table represent same virus species in strains of *Leptosphaeria biglobosa* and *Botrytis cinerea* isolated from co-infection samples.

Supplementary Table S6. Assembled sequences with similarity to previously described viruses in *Botrytis cinerea* from the co-infection samples

| Number | Contig number | Number of reads | Contig length | GenBank accesion numbers | Name of putative viruses | Best match | aa identity (%) | Genome type | Family/Genus |
|---|---|---|---|---|---|---|---|---|---|
| 1 | Contig 1 | 2479 | 10061 | OL602084 | Botrytis cinerea hypovirus 1 (BcHV1) | QBA69887.1 polyprotein [Botrytis cinerea hypovirus 1] | 99.8 | dsRNA | *Hypoviridae* |
| 2 | Contig 2 | 52362 | 9900 | OL602085 | Botrytis cinerea negative-stranded RNA virus 7 (BcNSRV7) | AWY11038.1 RNA-dependent RNA polymerase, partial [Sclerotinia sclerotiorum negative-stranded RNA virus 5] | 99.3 | -ssRNA | *Peribunyaviridae* |
| 3 | Contig 4 | 5972 | 7745 | OL602086 | Botrytis cinerea negative-stranded RNA virus 12 (BcNSRV12) | QKW91262.1 RNA dependent RNA polymerase [Botrytis cinerea negative-stranded RNA virus 8] | 76 | -ssRNA | unclassified |
| 4 | Contig 5 | 3581 | 7282 | OL602087 | Botrytis cinerea mycotymovirus 1 (BcMTV1) | QOE77941.1 replication-associated polyprotein [Sclerotinia sclerotiorum mycotymovirus 2] | 97.5 | +ssRNA | *Tymoviridae* |
| 5 | Contig 6 | 4606 | 6866 | OL602088 | Botrytis cinerea deltaflexivirus 2 (BcDFV2) | YP_009552771.1 polyprotein [Sclerotinia sclerotiorum deltaflexivirus 2] | 95.8 | +ssRNA | *Deltaflexiviridae* |
| 6 | Contig 8 | 838 | 5575 | OL602089 | Botrytis cinerea deltaflexivirus 1 (BcDFV1) | QJT73731 replication-associated polyprotein [Botrytis cinerea deltaflexivirus 1] | 98.8 | +ssRNA | *Deltaflexiviridae* |
| 7 | Contig 11 | 43387 | 4752 | OL602090 | Botrytis virus F (BV-F) | AWV68780.1 replicase [Botrytis virus F] | 96.1 | +ssRNA | *Gammaflexiviridae* |
| 8 | Contig 12 | 22294 | 4467 | OL602091 | Botrytis cinerea mitovirus 3 (BcMV3) | CEZ26302.1 RNA dependent RNA polymerase [Botrytis cinerea mitovirus 3] | 93.5 | +ssRNA | *Mitoviridae* |
| 9 | Contig 17 | 26757 | 3919 | OL602092 | Botrytis cinerea ourmia-like virus 2 (BcOLV2) | QJT73668 RNA-dependent RNA polymerase [Botrytis cinerea ourmia-like virus 2] | 45.5 | +ssRNA | *Botourmiaviridae* |
| 10 | Contig 23 | 73747 | 3696 | OL602093 | Botrytis cinerea umbra-like virus 2 (BcUV2) | AWY11003.1 RNA-dependent RNA polymerase [Sclerotinia sclerotiorum umbra-like virus 3] | 96 | +ssRNA | *Tombusviridae* |
| 11 | Contig 41 | 165290 | 2977 | OL602094 | Botrytis cinerea ourmia-like virus 12 (BcOLV12) | QJT73678 RNA-dependent RNA polymerase [Botrytis cinerea ourmia-like virus 12] | 95.9 | +ssRNA | *Botourmiaviridae* |
| 12 | Contig 46 | 467314 | 2655 | OL602095 | Botrytis cinerea mitovirus 7 (BcMV7) | YP_009182164.1 RNA dependent RNA polymerase [Sclerotinia sclerotiorum mitovirus 3] | 96.9 | +ssRNA | *Mitoviridae* |

| | | | | | | | | | |
|---|---|---|---|---|---|---|---|---|---|
| 13 | Contig 52 | 15889 | 2557 | OL602096 | Botrytis cinerea binarnavirus 1 (BcBNV1) | QJT73724 RNA-dependent RNA polymerase [Botrytis cinerea binarnavirus 1] | 94.9 | +ssRNA | *Narnaviridae* |
| 14 | Contig 53 | 10748 | 2552 | OL602097 | Botrytis cinerea deltaflexivirus 3 (BcDFV3) | QYU59136 hypothetical protein [Sclerotinia sclerotiorum deltaflexivirus 3S2] | 98.4 | +ssRNA | *Deltaflexiviridae* |
| 15 | Contig 66 | 104755 | 2398 | OL602098 | Botrytis cinerea ourmia-like virus 5 (BcOLV5) | QJT73671 RNA-dependent RNA polymerase [Botrytis cinerea ourmia-like virus 5] | 97.2 | +ssRNA | *Botourmiaviridae* |
| 16 | Contig 93 | 36070 | 2190 | OL602099 | Botrytis cinerea ourmia-like virus 9 (BcOLV9) | QJT73675 RNA-dependent RNA polymerase [Botrytis cinerea ourmia-like virus 9] | 96.4 | +ssRNA | *Botourmiaviridae* |
| 17 | Contig 104 | 4866 | 2134 | OL602100 | Botrytis cinerea binarnavirus 6 (BcBNV6) | AXE72934.1 RNA-dependent RNA polymerase [Aspergillus fumigatus narnavirus 2] | 46.8 | +ssRNA | *Narnaviridae* |
| 18 | Contig 202 | 118 | 1836 | OL602101 | Botrytis cinerea RNA virus 2 (BcRV2) | QBA69891.1 RNA-dependent RNA polymerase [Botrytis cinerea RNA virus 2] | 98.2 | dsRNA | unclassified |
| 19 | Contig 465 | 399 | 1504 | OL602102 | Botrytis cinerea partitivirus 4 (BcPV4) | YP_001686790.1 coat protein [Botryotinia fuckeliana partitivirus 1] | 95.8 | dsRNA | *Partitiviridae* |
| 20 | Contig 1241 | 147491 | 1134 | OL602103 | Botrytis cinerea mitovirus 4 (BcMV4) | YP_009182163.1 RNA dependent RNA polymerase [Botrytis cinerea mitovirus 4] | 95.8 | +ssRNA | *Mitoviridae* |
| 21 | Contig 1314 | 19311 | 1120 | OL602104 | Botrytis cinerea partitivirus 2 (BcPV2) | QBA69895.1 coat protein [Botrytis cinerea partitivirus 2] | 99.4 | dsRNA | *Partitiviridae* |
| 22 | Contig 1409 | 170 | 1092 | OL602105 | Botrytis cinerea mycovirus 5 (BcMyV5) | QJT73712 RNA-dependent RNA polymerase [Botrytis cinerea mycovirus 5] | 99.5 | dsRNA | unclassified |
| 23 | Contig 4602 | 25 | 703 | OL602106 | Botrytis cinerea tetramycovirus-1 (BctRV1) | AWY10947.1 methyltransferase [Sclerotinia sclerotiorum tetramycovirus-1] | 73.5 | dsRNA | unclassified |
| 24 | Contig 5584 | 55881 | 642 | OL602107 | Botrytis cinerea umbra-like virus 1 (BcUV1) | MN625251.1 RNA-dependent RNA polymerase [ Botrytis cinerea umbra-like virus 1] | 90.1 | +ssRNA | *Tombusviridae* |
| 25 | Contig 7010 | 22 | 567 | OL602108 | Botrytis cinerea endornavirus 3 (BcEV3) | QLC37097.1 polyprotein [Botrytis cinerea endornavirus 3] | 95.2 | dsRNA | *Endornaviridae* |

Note: The grey shading filled areas in the table represent same virus species in strains of *Leptosphaeria biglobosa* and *Botrytis cinerea* isolated from co-infection samples.

Supplementary Table S7. Oligonucleotide primers used for revere transcription (RT)-PCR detection of mycoviruses in *Leptosphaeria biglobosa* and *Botrytis cinerea*

| Primer name | Sequence (5'→3') | Size | Polarity |
|---|---|---|---|
| LbMV3-R | TCTAACTTTCCCAGCTGCCTC | 732 | - |
| LbMV3-F | AGGGTCGAGTCAGTAGGATTCA | | + |
| LbNSRV3-R | GGGGCACTCAGAACTGTCAA | 873 | - |
| LbNSRV3-F | AGGACACCTGCACAACACAA | | + |
| BcUV1-R | AGTCTAAGGCCGTACAGGAG | 430 | - |
| BcUV1-F | CGACCCTCTCTAGGGACCAC | | + |
| BcMV7-R | ACGTTGTCCTCCTAAATCCCG | 760 | - |
| BcMV7-F | CTCTGTAGGTCAACCGATGG | | + |
| BVF-R | CTGCTCCCAGCAATGACAAG | 698 | - |
| BVF-F | AGTGGTGGGCAATCCAAGTG | | + |
| BcMV4-R | ACCGGAAGCATAGAATCGTGT | 753 | - |
| BcMV4-F | TGGGATAGAGGTTAGTGGGATT | | + |
| BcMV4B-F | TTCAACTTTAACAATTGTACCGGA | 844 | + |
| BcMV4B-R | GATGGGATAGAGGTTAGTGGGA | | - |
| BcMV4S-F | TTCAACTTTAACAATTGTACCGGA | 494 | + |
| BcMV4S-R | TGGTGGGATGGTGTCTGAAG | | - |
| BcBNV1-F | GCGTAAGATGGGTGGGATGC | 852 | + |
| BcBNV1-R | GGGCACCGCTACGATTTTC | | - |
| BcBNV6-F | ACTCGGACGGGATATAACGG | 741 | + |
| BcBNV6-R | GACTTCACAGCGGGACCAATA | | - |
| BcDFV1-F | TCATGGCGTGAGAAAGCGG | 808 | + |
| BcDFV1-R | TCGTCACCATGGATGCACTAC | | - |
| BcMyV5-F | CTGAGGAACTTGCCGAGGTA | 644 | + |
| BcMyV5-R | ACCAACGCCTATTGTGGAGA | | - |

Supplement to Supplementary Table S7

| Primer name | Sequence (5’→3’) | Size | Polarity |
|---|---|---|---|
| BcEV3-F | ACGCATTTGCCTCTAGCCTG | 344 | + |
| BcEV3-R | CACGGACGATGATTTTGACGG | | - |
| BcHV1-F | TGCAAGTTCTACCAGTTCTTCCT | 996 | + |
| BcHV1-R | TTGGTGGTCTAAAGGGTGGT | | - |
| BcMV3-F | GAAATCTGGCTGGTCTCCGA | 993 | + |
| BcMV3-R | GATCTGCCGAGCTAATTATAGCC | | - |
| BcMTV1-F | AACTTCTGAATCCCTCCCGC | 957 | + |
| BcMTV1-R | GGCAGGTTCCACAGTCCAAG | | - |
| BcNSRV7-F | GCTGATGCTCAATATCCGGG | 811 | + |
| BcNSRV7-R | TGAGCCATTCAAAGGGGATTCT | | - |
| BcOLV2-F | TCTGCGAGAGCCATATCCAC | 784 | + |
| BcOLV2-R | GACCACTCTCCCACTCACTTG | | - |
| BcOLV5-F | ATGAAACGAGCGGTACGGG | 902 | + |
| BcOLV5-R | TCTGACGACCACTGAACGC | | - |
| BcPV2-F | CTGGAGCCACTGGAAACGTC | 703 | + |
| BcPV2-R | CGCGAACGACCACCAAGATT | | - |
| BcPV4-F | CACCAGCAGAAGGTTTGTCC | 904 | + |
| BcPV4-R | CGAAAGTCCAAACCTGGCAA | | - |
| BcRV2-F | TCAACGAAGCTGAAGGCACT | 885 | + |
| BcRV2-R | TTCGTTGATCCATCCCTCGG | | - |
| BctRV1-F | CGTCATTAGCGAGGAGCGAT | 448 | + |
| BctRV1-R | GAGAGTGGAAAGCTCCGGTC | | - |
| LbMV3-F | GGAACGAATCCAAAAGGACCC | 976 | + |
| LbMV3-R | GGTCACAGAATCCTCTCTTCCC | | - |
| LbNV1-F | TGAGCCGTCCTCTATTCTGGA | 715 | + |
| LbNV1-R | GTGAACCTCAAACCACGACG | | - |
| LbDFV1-F | AAGCTTTGGTTCGCTGCCAC | 771 | + |
| LbDFV1-R | AAAAAGGAAATGGTTGCGCCC | | - |

Supplementary Table S8. Presence of different mycoviruses in *Leptosphaeria biglobosa* and *Botrytis cinerea* strains.

| Species | Strains | Species of mycovirus | | | | | |
|---|---|---|---|---|---|---|---|
| | | BcMV4 | BcUV1 | BVF | LbNSRV3 | LbMV3 | BcMV7 |
| *B. cinerea* | RCT-6 | - | + | - | - | - | - |
| | RCT-7 | + | + | + | - | - | - |
| | WCT-3 | - | - | + | - | - | - |
| | WCT-4 | + | - | + | - | - | + |
| | ZC-7 | - | - | + | - | - | + |
| | ZC-8 | + | - | + | - | - | + |
| | ZC-9 | + | - | + | - | - | + |
| | ZC-10 | - | - | - | - | - | - |
| | HC-3 | - | - | + | - | - | - |
| | WH-1 | + | + | - | - | - | - |
| | WH-3 | - | - | - | - | - | - |
| | WH-5 | - | - | - | - | - | - |
| | WH-6 | - | - | - | - | - | - |
| | WH-11 | + | - | + | - | - | - |
| | HYJ-5 | - | - | + | - | - | - |
| | WHG-5 | - | - | + | - | - | - |
| *L. biglobosa* | RCT-4 | - | + | - | - | - | - |
| | WCT-2 | - | + | - | - | - | - |
| | WCT-6 | - | + | - | - | - | - |
| | WCT-7 | - | + | - | + | - | - |
| | WCT-8 | + | - | - | + | - | - |
| | WCT-9 | - | + | - | - | - | - |
| | WCT-10 | - | - | - | - | - | - |
| | HC-1 | - | - | - | - | - | - |
| | ZC-2 | - | + | - | - | + | - |
| | ZC-3 | + | + | - | - | - | - |
| | WH-2 | + | - | - | - | - | - |
| | WH-4 | - | + | - | - | - | - |
| | WH-10 | + | - | - | - | - | - |
| | QC-5 | - | - | - | - | - | - |
| | QC-6 | + | - | - | - | + | - |

Note that "+" and " - " represents the presence and non-presence of the corresponding virus in fungal strains through the detection of RT-PCR with primer pairs listed in Supplementary Table 7.
